# Unprecedented lattice volume expansion on doping stereochemically active Pb^2+^ into uniaxially strained structure of CaBa_1−*x*_Pb_*x*_Zn_2_Ga_2_O_7_

**DOI:** 10.1038/s41467-020-14759-2

**Published:** 2020-03-11

**Authors:** Pengfei Jiang, Joerg C. Neuefeind, Maxim Avdeev, Qingzhen Huang, Mufei Yue, Xiaoyan Yang, Rihong Cong, Tao Yang

**Affiliations:** 1 College of Chemistry and Chemical Engineering, Chongqing University, Chongqing, 401331 P. R. China; 2 Chemical and Engineering Materials Division, Spallation Neutron Source, Oak Ridge National Laboratory, Oak Ridge, TN 37831 USA; 3 Australian Nuclear Science and Technology Organization, Lucas Heights, NSW 2234 Australia; 4 School of Chemistry, The University of Sydney, Sydney, NSW 2006 Australia; 5 NIST Center for Neutron Research, National Institute of Standards and Technology, Gaithersburg, MD 20899 USA; 6 College of Materials Science and Engineering, Guilin University of Technology, Guilin, Guangxi 541004 P. R. China

**Keywords:** Chemistry, Physics

## Abstract

Lone pair cations like Pb^2+^ are extensively utilized to modify and tune physical properties, such as nonlinear optical property and ferroelectricity, of some specific structures owing to their preference to adopt a local distorted coordination environment. Here we report that the incorporation of Pb^2+^ into the polar “114”-type structure of CaBaZn_2_Ga_2_O_7_ leads to an unexpected cell volume expansion of CaBa_1-*x*_Pb_*x*_Zn_2_Ga_2_O_7_ (0 ≤ *x* ≤ 1), which is a unique structural phenomenon in solid state chemistry. Structure refinements against neutron diffraction and total scattering data and theoretical calculations demonstrate that the unusual evolution of the unit cell for CaBa_1-*x*_Pb_*x*_Zn_2_Ga_2_O_7_ is due to the combination of the high stereochemical activity of Pb^2+^ with the extremely strained [Zn_2_Ga_2_O_7_]^4−^ framework along the *c*-axis. The unprecedented cell volume expansion of the CaBa_1−*x*_Pb_*x*_Zn_2_Ga_2_O_7_ solid solution in fact is a macroscopic performance of the release of uniaxial strain along *c*-axis when Ba^2+^ is replaced with smaller Pb^2+^.

## Abstract (web summary)

Lone pair cations can impart interesting features in some structures, such as noncentrosymmetry. Here the authors show unexpected cell volume expansion in a polar “114”-type oxide upon replacing Ba^2+^ with a smaller Pb^2+^, and attribute it to high stereochemical activity of Pb^2+^ with the strained framework.

## Introduction

Lone pair (LP) cations (Tl^+^, Pb^2+^, Bi^3+^, Te^4+^, Sb^3+^, I^5+^, etc.) with (n−1)*d*^10^n*s*^2^ electronic configurations are prone to adopt an asymmetric coordination environment and thus form a locally distorted structural unit possessing a dipolar moment. A long-range ordered alignment of such asymmetric units may break the inversion symmetry of the structure, possibly leading to a polar structure with intriguing properties. The strategy of using asymmetric units with LP cations is extensively utilized to design and synthesize noncentrosymmetric structures with enhanced nonlinear optical properties^1–7^. Moreover, LP cations in polar structures may lead to spontaneous electric polarizations and form ferroelectric structure, e.g., BiFeO_3_^8^. In some cases, such LP cations-induced ferroelectricity can also trigger large negative thermal expansion (NTE) upon warming, which has also been extensively investigated in PbTiO_3_-based perovskites^9–13^.

Recently, the stereochemical activity owing to the LP electrons of Sn^2+^ in CsSnBr_3_^14^, and Pb^2+^ and Sn^2+^ in rock-salt chalcogenides PbS^15^, PbTe^15^, and SnTe^16^, is shown to cause a local symmetry lowering in a limited temperature range upon warming. This phenomenon is unique because it is an opposite behavior to that of most crystals showing symmetry lowering on cooling. X-ray and neutron total scattering experiments revealed that the progressively local distortion state in these compounds stems from the lattice expansion-induced dynamic off-center displacement of LP cations upon warming^14,15^.

As described above, the incorporation of the stereochemically active LP cations into some structures brings not only intriguing physical properties but also some uncommon phenomena. In this work, we report another unprecedented phenomenon based on stereochemical activity of LP cations, i.e., cell volume expansion in solid solutions of CaBa_1−*x*_Pb_*x*_Zn_2_Ga_2_O_7_ (0 ≤ *x* ≤ 1) when substituting Ba^2+^ (*r* = 1.61 Å in 12-fold coordination) with smaller Pb^2+^ (*r* = 1.49 Å in 12-fold coordination)^17^. Ostensibly, the abnormal cell volume expansion in CaBa_1−*x*_Pb_*x*_Zn_2_Ga_2_O_7_ is ascribed to the fact that the expansion of the *c* axis (0.11 Å) is much larger than the contraction of the *a* axis length (0.011 Å) in the space group *P*6_3_*mc*. Neutron pair distribution function (nPDF) data analyses confirm that CaBa_1−*x*_Pb_*x*_Zn_2_Ga_2_O_7_ locally adopt a distorted orthorhombic structure (*Pna*2_1_); however, this local distortion is not responsible for the abnormal cell volume expansion, as suggested by the Rietveld refinements based on high-resolution X-ray and neutron diffraction (ND) data. Density functional theory (DFT) calculations reveal that Pb^2+^ 6*s*6*p* orbitals are highly hybridized with O^2−^ 2*p* orbitals, which leads to the formation of a strong covalent bond and the resulting structural strain of the original [Zn_2_Ga_2_O_7_]^4−^ framework is released by significantly elongating the *c* axis length.

## Results

### Unexpected cell volume expansion for CaBa_1−__*x*_Pb_*x*_Zn_2_Ga_2_O_7_

CaBa_1−*x*_Pb_*x*_Zn_2_Ga_2_O_7_ crystallize in the so-called “114”-type oxide structure, where the [BaO_3_] and [O_4_] layers form a mixed cubic and hexagonal closed-packing ionic structure with the stacking sequence of -CBABC-, leaving the octahedral and tetrahedral cavities partially occupied by Ca^2+^ and Zn^2+^/Ga^3+^ cations, respectively^18^. This structure is more commonly viewed as a layered structure with alternating stacking of triangular and Kagome layers along the polar axis. Up to now, nitrogen ions and various metal cations could be incorporated into this structure^19–23^. This work is the report of Pb^2+^ doping.

**Fig. 1:**
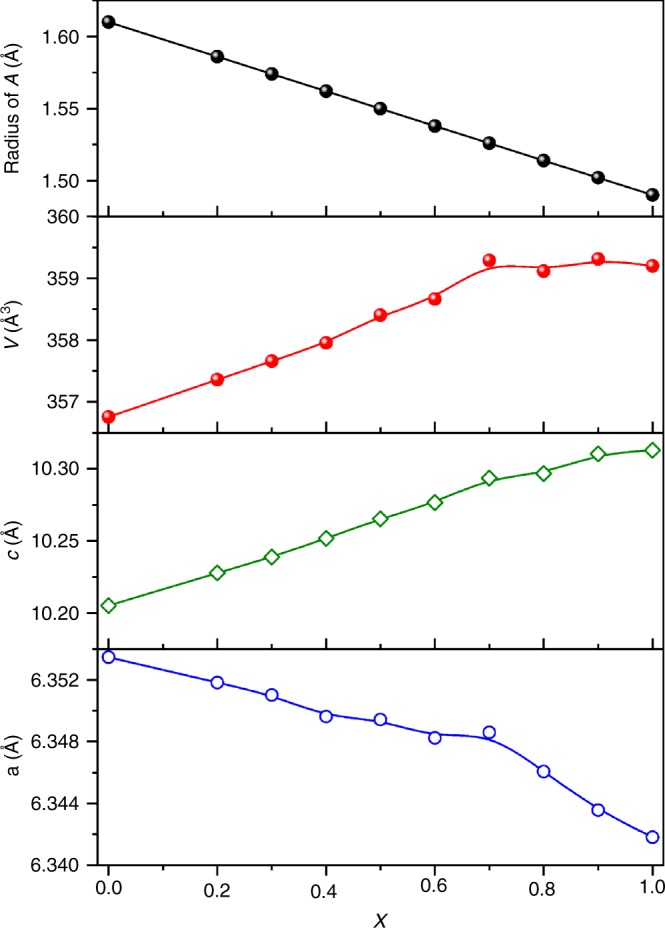
Lattice parameters. Plots of the average A-site size and refined lattice parameters for CaBa_1−*x*_Pb_*x*_Zn_2_Ga_2_O_7_ along with the increasing Pb content. The average radius of *A*-site cation is calculated according to the equation: *r*(A^2+^) = (1−*x*) *r*(Ba^2+^) + *x r*(Pb^2+^). Source data are provided as a Source Data file.

Powder X-ray diffraction (XRD) patterns for CaBa_1−__*x*_Pb_*x*_Zn_2_Ga_2_O_7_ (Supplementary Fig. 1) indicate the phase purity as well as the high crystallinity of the samples. The anisotropic change of the lattice parameters can be readily visualized from XRD, as the position for the (004) reflection evolves to lower angles, whereas the position for the (200) reflection shifts to higher angles on the Pb^2+^-to-Ba^2+^ substitutions. It is firmly corroborated by plotting the lattice parameters against the Pb^2+^ content (Fig. 1), where the unit cell lattice contracts and expands along the *a* and *c-*axes, respectively. Such an anisotropic change leads to an overall expansion of the unit cell volume for CaBa_1−__*x*_Pb_*x*_Zn_2_Ga_2_O_7_ up to *x* = 0.7 and saturates thereafter. As discussed above, given the smaller ionic radius of Pb^2+^ compared with that of Ba^2+^, this is an unexpected and unique structural phenomenon in solid state chemistry. One might argue that the simple comparison of the cationic radii for Ba^2+^ and Pb^2+^ is not sufficient because the ionic size for LP cation-like Pb^2+^ is not well defined owing to their typical distorted coordination environment. Therefore, a series of Ba^2+^- and Pb^2+^-containing compounds with identical structure types were compared in terms of their cell volumes per formula, as summarized in Supplementary Table 1. It is obvious that all the Pb^2+^-containing compounds possess smaller volumes in comparison with those of Ba^2+^-containing compounds, which confirms that the observed cell volume expansion in CaBa_1−__*x*_Pb_*x*_Zn_2_Ga_2_O_7_ is unprecedented.

Furthermore, we synthesized CaBa_0.5_Sr_0.5_Zn_2_Ga_2_O_7_ for comparison, because Sr^2+^ (*r* = 1.44 Å) has a comparable cationic radius with Pb^2+^^17^, however, the Sr^2+^-to-Ba^2+^ substitution led to a significant cell volume contraction, i.e., V = 351.55 Å^3^ for CaBa_0.5_Sr_0.5_Zn_2_Ga_2_O_7_ and 356.73 Å^3^ for CaBaZn_2_Ga_2_O_7_. Accordingly, we conclude that it is not the cationic size that governs the lattice expansion in CaBa_1−*x*_Pb_*x*_Zn_2_Ga_2_O_7_, instead, it strongly points to the special characteristics of Pb^2+^ (6 *s*^2^ LP electrons).

### Reciprocal space structure refinements

Crystal structures for all solid solutions were investigated by Rietveld refinements using XRD data so as to reveal the origin of this unexpected cell volume expansion. CaBaZn_2_Ga_2_O_7_ was used as the starting structural model (*P*6_3_*mc*) for structure refinements. Zn^2+^ and Ga^3+^ could not be distinguished by X-ray scattering owing to their similar atomic form factors, thus Zn^2+^ and Ga^3+^ were treated as the same cation during the Rietveld refinements on XRD. As all the samples were found phase pure and metal disorder between (Ba, Pb) and (Ga, Zn) sites is implausible due to very large difference in size (>220%), the occupancy factors for Ba^2+^ and Pb^2+^ were fixed to the nominal values. Finally, a model with anisotropic atomic displacements (ADPs) was used to account for the local displacement disorder Ba^2+^/Pb^2+^ cations in CaBa_1−*x*_Pb_*x*_Zn_2_Ga_2_O_7_ (0 < *x* < 1) (see more detail in Supplementary Note 2). The ADPs for the (Ba/Pb) site were found elongated along the *c* axis (Supplementary Table 2), which is consistent with the LP effect of Pb^2+^. Moreover, the U_33_/U_11_ ratio evolution with *x* proved a good indicator of the structural strain being relieved with increasing content of Pb^2+^. At low doping levels, where the geometry of the crystal structure is determined mostly by Ba^2+^, the local displacements mimicked by the ADPs are the largest (Supplementary Fig. 2). Further increasing content of Pb^2+^ relieves the strain by stretching the *c* axis and the ADPs become progressively isotropic. In the composition with the highest *x*, it is Pb^2+^ that controls the crystal structure geometry and *c* axis becomes too long for Ba^2+^ and ADP becomes a flattened ellipsoid within the *ab*-plane, so CaPbZn_2_Ga_2_O_7_ was described with a site splitting model. The refinements converged rapidly to give stable structures with reasonable crystallographic parameters and advantageous agreement factors for the solid solutions. The final Rietveld refinement patterns for solid solutions are presented in Supplementary Fig. 3. The resultant crystallographic data are summarized in Supplementary Tables 2 and 3.

To determine the crystal structure accurately, high-resolution constant wavelength ND data and Cu Kα1 data for representative compositions CaBa_1−*x*_Pb_*x*_Zn_2_Ga_2_O_7_ (*x* = 0, 0.5, and 1) were collected. By ND, the occupancy factors of Ga^3+^ and Zn^2+^ at T sites could be determined, because there is a large contrast in neutron scattering length between Zn^2+^ (5.68 fm) and Ga^3+^ (7.29 fm). Combined Rietveld refinements against both the ND and XRD data revealed that the occupancy factor for Zn^2+^ at T1 site converged to ~ 0.21 for all compositions, which confirms that the evolution of cell parameters and cell volume as a function of *x* is driven by Pb^2+^ content, and not by Zn/Ga distribution, which remains constant across the series.

**Fig. 2:**
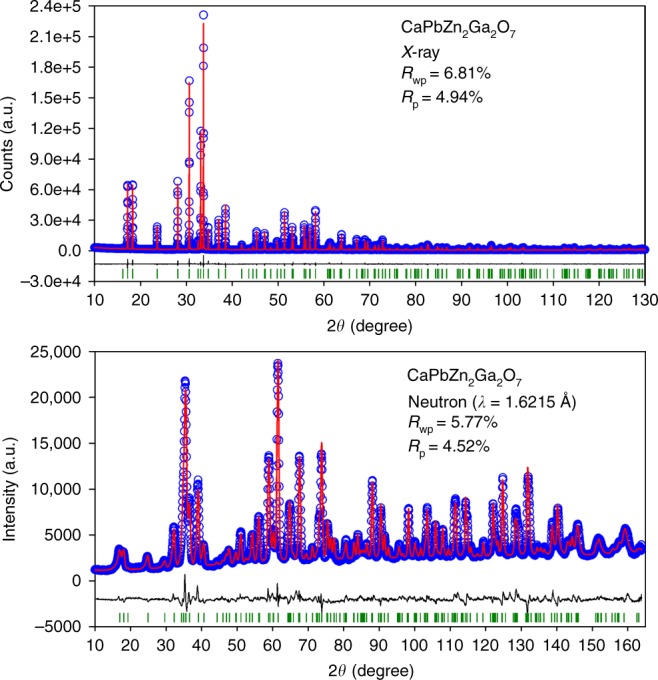
Reciprocal space structure refinements. Combined Rietveld refinement plots of Cu Kα_1_ XRD and constant wavelength ND data for CaPbZn_2_Ga_2_O_7_ refined with the *P*6_3_*mc* model. The overall reliability factors for combined Rietveld refinements are *R*_wp_ = 6.44%, *R*_p_ = 4.77%. Source data are provided as a Source Data file.

It is noteworthy that the local displacement of Pb^2+^ does not cause any average structure symmetry lowering and the CaBa_1−*x*_Pb_*x*_Zn_2_Ga_2_O_7_ samples can be well described using *P*6_3_*mc*. All these observations in CaBa_1−*x*_Pb_*x*_Zn_2_Ga_2_O_7_ are thus different from other isostructural oxides without stereochemically active cations, e.g., *MA*Zn_2_Ga_2_O_7_ (*M* = Ca^2+^, Sr^2+^; *A* = Ba^2+^, Sr^2+^), where successive symmetry lowering and cation disorder-order transitions were observed^18^. Such symmetry and cation disorder-order transitions might be observed for CaBa_1−*x*_Pb_*x*_Zn_2_Ga_2_O_7_ at lower temperatures. The plots of combined Rietveld refinements are presented in Fig. 2 and Supplementary Fig. 4. The final crystallographic data and selected interatomic distances are summarized in Supplementary Tables 4 and 5.

### Real space structure refinements

Combined Rietveld refinements using both XRD and ND data are powerful to analyze the average structure. On the other hand, the total scattering technology allows both the coherent and diffuse components of the XRD/ND pattern to be properly accounted for when modeling a crystal structure. We utilized nPDF analyses to reveal the evolution of local structural distortions and cationic ordering, which helps understand the unique cell volume expansion phenomenon in CaBa_1−*x*_Pb_*x*_Zn_2_Ga_2_O_7_.

The normalized structure functions and nPDFs for CaBa_1−*x*_Pb_*x*_Zn_2_Ga_2_O_7_ (*x* = 0, 0.5, and 1) are presented in Supplementary Fig. 5. Apparent shift of the positions of Pb/Ba−O and Zn/Ga−Zn/Ga pairs are observed for the nPDFs (Supplementary Fig. 5b), which is in line with the results deduced from XRD and ND. The peak for nearest Zn/Ga−O pairs is almost symmetric although the cationic size difference for Zn^2+^ and Ga^3+^ is large. Moreover, owing to the overlap of the positions between Zn/Ga−Zn/Ga and Pb/Ba−O pairs, the local Zn/Ga ordering cannot be visually observed. Apart from the change of peak positions, another noteworthy feature is the obvious decrease or increase in the magnitude of the atomic pairs with increasing Pb^2+^ content, i.e., Zn/Ga−O and Ca−O pairs, indicating a wide range of atomic distributions. This observation suggests the Pb^2+^ doping may lead to a local structure distortion.

**Fig. 3:**
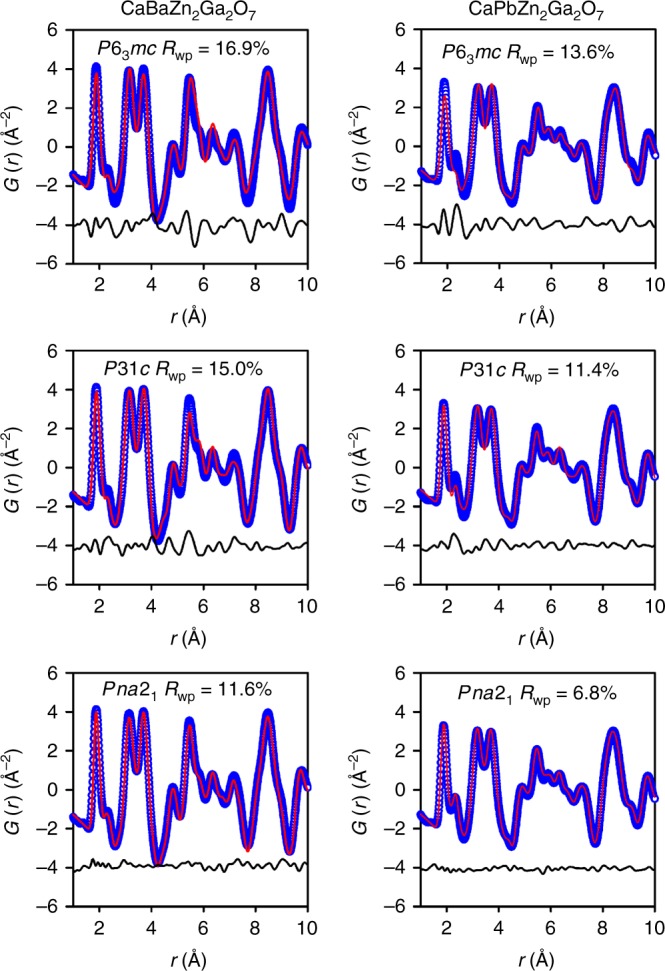
Real space structure refinements. Real space Rietveld refinements of neutron PDF data for CaBaZn_2_Ga_2_O_7_ and CaPbZn_2_Ga_2_O_7_ with *P*6_3_*mc*, *P*31*c,* and *Pna*2_1_ models. Source data are provided as a Source Data file.

Then real space refinements were performed against the neutron PDF data with the average structure model (*P*6_3_*mc*) obtained from the combined refinement. However, this model does not reproduce well the peak at ~ 2.3 Å, which is corresponding to the Ca−O pairs (Fig. 3 and Supplementary Fig. 6). Moreover, this structure model also overestimates the Zn/Ga−O distances (at ~ 4.8 and 6.3 Å) by ~ 0.08 Å. These observations suggest that neither the CaO_6_ nor (Zn/Ga)O_4_ polyhedra extracted from the neutron PDF data can be well described by the average structure model, especially in the low *r* range. This is a strong indication of the local structural distortion in CaBa_1−*x*_Pb_*x*_Zn_2_Ga_2_O_7_. To gain an accurate picture of the local structure, structure models in the space group *P*31*c* and *Pna*2_1_, which allow the free distortion of (Zn/Ga)O_4_ tetrahedra, were employed. As shown in Fig. 3 and Supplementary Fig. 6, the refinement using the *P*31*c* model gives agreement similar to that of the *P*6_3_*mc* model, whereas the refinements using the *Pna*2_1_ model provides an excellent fit for both peaks of Ca−O and Zn/Ga−O pairs. Therefore, the local structure for CaBa_1−*x*_Pb_*x*_Zn_2_Ga_2_O_7_ exhibits a lower symmetry, *Pna*2_1_. Because both Pb^2+^-containing and -free compounds are locally distorted, such a local structure distortion unambiguously ascribes to Zn^2+^/Ga^3+^ disordering, rather than the Pb^2+^-to-Ba^2+^ substitutions. It was recently reported that a similar cation inversion disordering induced a local structure distortion in spinel Mg_1−*x*_Ni_*x*_Al_2_O_4_ and CuMn_2_O_4_^24,25^, which were also probed by the neutron total scattering technique. Here, the refined nanoscale structures for CaBa_1−*x*_Pb_*x*_Zn_2_Ga_2_O_7_ (*x* = 0 and 1) (Supplementary Fig. 7) demonstrate that the local orthorhombic distortion is not significant, further indicating the distortion on the long range would be also insignificant. The lattice parameters extracted from neutron PDF analyses also exhibit an anisotropic shrinkage and expansion within the *ab*-plane and along the *c* axis (Supplementary Fig. 8), respectively, which results in the increase of the unit cell volume with the increasing Pb^2+^ content.

Combined reciprocal and real space Rietveld refinements against ND and PDF data were further performed to reveal the long-range structure symmetry. In the long range, only the *P*6_3_*mc* and *P*31*c* models were considered because the reflection conditions for the *Pna*2_1_ model are not consistent with the ND data. As shown Supplementary Figs. 9–11, all the PDF peaks for CaBa_1−*x*_Pb_*x*_Zn_2_Ga_2_O_7_ (*x* = 0, 0.5, 1) in long range (30–50 Å) can be well produced by the *P*6_3_*mc* model with reliable factors comparable with or even better than that of the low symmetry *P*31*c* model, demonstrating the long-range structure symmetry for CaBa_1−*x*_Pb_*x*_Zn_2_Ga_2_O_7_ is *P*6_3_*mc*, which is in good agreement with the combined Rietveld analysis of XRD and ND data.

### Structure evolution

**Fig. 4:**
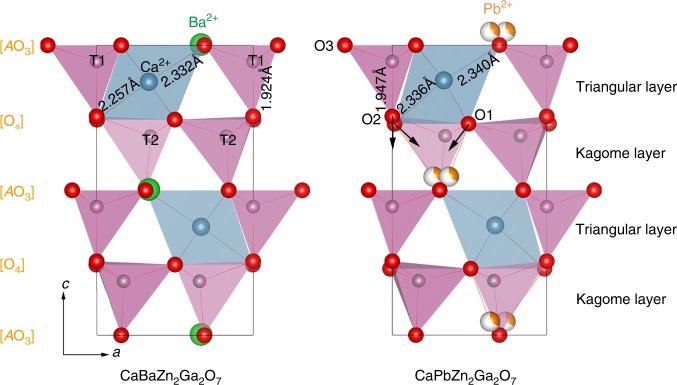
Crystal structures for CaBaZn_2_Ga2O_7_ and CaPbZn_2_Ga_2_O_7_. Crystal structures were obtained from the combined Rietveld refinements on ND and XRD data with the *P*6_3_*mc* model. In both structures T1 and T2 sites are co-occupied by Ga^3+^ and Zn^2+^ but with different occupancies, where T1 site is dominated by Ga^3+^ with an occupancy factor of 0.79(3), accordingly T2 site is mainly occupied by Zn^2+^ with an occupancy factor of 0.60(2). The black arrows represent the shift direction of oxygen atoms.

**Fig. 5:**
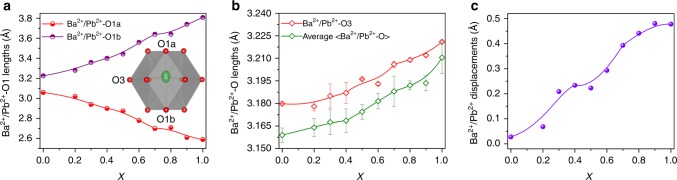
Evolution of Ba/Pb−O bond lengths and Ba/Pb displacements. Plots of Pb^2+^/Ba^2+^−O1 bond lengths **a**, Pb^2+^/Ba^2+^−O3 and average < Pb^2+^/Ba^2+^−O > bond lengths **b**, and average Pb^2+^/Ba^2+^ displacement distance **c** against the Pb content in CaBa_1−*x*_Pb_*x*_Zn_2_Ga_2_O_7_. The inset shows the coordination environment of Ba^2+^/Pb^2+^ in CaBa_1−*x*_Pb_*x*_Zn_2_Ga_2_O_7_ (0 < *x* < 1) obtained from Rietveld refinements against XRD data with the *P*6_3_*mc* model, where a highly anisotropic thermal motion along *c* axis can be visually observed. For *x* = 0, 0.5, and 1, the parameters are obtained from combined Rietveld refinement against both XRD and ND data. Source data are provided as a Source Data file.

Figure 4 and Supplementary Fig. 12 give the comparison crystal structures for CaBa_1−*x*_Pb_*x*_Zn_2_Ga_2_O_7_ (*x* = 0 and 1) and CaBa_0.5_Sr_0.5_Zn_2_Ga_2_O_7_. Apparently, Ba^2+^/Sr^2+^ in CaBaZn_2_Ga_2_O_7_ and CaBa_0.5_Sr_0.5_Zn_2_Ga_2_O_7_ are located in the same *ab*-plane defined by O3 anions. In contrast, the Pb^2+^ cations in CaPbZn_2_Ga_2_O_7_ show stereochemical activity with a significant displacement along the polar *c* axis, which can be also deduced from the change of Ba^2+^/Pb^2+^−O interatomic distances in CaBa_1−*x*_Pb_*x*_Zn_2_Ga_2_O_7_ (0 ≤ *x* ≤ 1). As shown in Fig. 5a, the difference between the group of Ba^2+^/Pb^2+^−O1 bond lengths becomes larger when increasing the Pb^2+^-content. The Ba^2+^/Pb^2+^−O3 bond also exhibits an increasing trend (Fig. 5b). Such evolution of Ba^2+^/Pb^2+^−O bond lengths indicates that the Pb^2+^ cation displaces from the center of the [Ba/PbO_12_] dodecahedron rather than merely attracts the apical oxygen atom closer as in the case of CaBa_0.5_Sr_0.5_Zn_2_Ga_2_O_7_ (Supplementary Table 3).

The calculated average deviation distance (*D*) for Ba^2+^/Pb^2+^ continuously increases along with the increase of the Pb^2+^ content (Fig. 5c). The largest *D* value ~ 0.5 Å is observed in CaPbZn_2_Ga_2_O_7_. Such a significant deviation of Pb^2+^ from the O3-plane towards to O1 results in a very irregular coordination environment for Pb^2+^. The shortest Pb−O bond length (2.337 Å) in CaPbZn_2_Ga_2_O_7_ is comparable with the values in Ca_2_PbGa_8_O_15_ (2.29 Å)^26^, PbTiO_3_ (2.51 Å)^27^, and PbRuO_3_ (2.50 Å)^28^, where the Pb 6*s*6*p* orbitals are all strongly hybridized with O 2*p* orbitals. So it is expected that the Pb^2+^ 6*s*6*p* orbitals in CaPbZn_2_Ga_2_O_7_ are also strongly hybridized with O 2*p* orbitals, which is further corroborated by DFT calculations. For example, the density of states analyses for CaPbZn_2_Ga_2_O_7_ (Supplementary Fig. 13) indicate that both the bottom of the conduction band and the top of the valence band comprise the Pb^2+^ 6*s*6*p* states, which is a solid evidence of the hybridization between Pb 6*s*6*p* and O 2*p* orbitals.

## Discussion

**Fig. 6:**
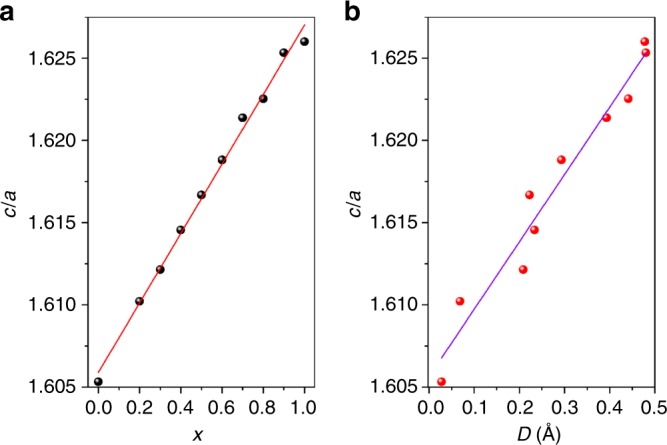
Evolution of *c/a* values. Plots of *c*/*a* values against **a** the doping Pb content and **b** the stereochemical lone pair activity (average displacements for Ba^2+^/Pb^2+^ cations) in CaBa_1−*x*_Pb_*x*_Zn_2_Ga_2_O_7_. For *x* = 0, 0.5, and 1, the parameters are obtained from combined Rietveld refinement against both XRD and ND data. Source data are provided as a Source Data file.

As discussed above, both real space and reciprocal data analysis decipher that the unprecedented cell volume expansion for CaBa_1−*x*_Pb_*x*_Zn_2_Ga_2_O_7_ (0 ≤ *x* ≤ 1) from nanoscale to a long-range scale is ascribed neither to the local structure distortion nor the cationic ordering. All these results deduced from the structure refinements point to the stereochemical activity of Pb^2+^. LP induced displacement along a polar axis is commonly observed for many Bi^3+^ and Pb^2+^-containing perovskites, i.e., Bi*M*O_3_^29–31^ and Pb*M*O_3_ (*M* = transition metals)^32,33^, which always results in a large tetragonality with *c*/*a* > 1.0. This enhanced tetragonality is closely related to the highly hybridization between Pb^2+^ 6 *s*^2^ and O^2−^ 2*p* orbitals, which is indeed observed in (*x*)BiFeO_3_-(1−*x*)PbTiO_3_^34^ and Pb_0.8-*x*_La_*x*_Bi_0.2_VO_3_^12^ solid solutions. Here in CaBa_1−*x*_Pb_*x*_Zn_2_Ga_2_O_7_, the enhancement of *c*/*a* values is also accompanied with the increase of the stereochemical activity of Ba/Pb^2+^ cations (or enhancement of Ba^2+^/Pb^2+^−O covalency) (Fig. 6a). This observation corroborates that the structural anisotropy as well as the cell volume expansion for CaBa_1−*x*_Pb_*x*_Zn_2_Ga_2_O_7_ is dominated by the stereochemically active LP electrons of Pb^2+^. Moreover, the *c*/*a* value for CaPbZn_2_Ga_2_O_7_ can be reduced upon heating (Supplementary Fig. 14), which is similar to the PbTiO_3_-based ferroelectricity-NTE materials^9,10^. This observation further reinforces the hypothesis that the stereochemical activity of Pb^2+^ is responsible for the anisotropic lattice expansion. However, NTE is not realized for CaPbZn_2_Ga_2_O_7_ and an expansion of the *c* axis rather than contraction was observed, suggesting the unique structure for “114” oxides should be also responsible for the lattice volume expansion.

To further elucidate this conjecture, we also prepared the solid solutions of Ba_1−*y*_Pb_*y*_TiO_3_ (*y* = 0, 0.5, and 1). Pb^2+^-to-Ba^2+^ substitutions in Ba_1−*y*_Pb_*y*_TiO_3_ also lead to an anisotropic change of the cell dimensions as expected, however the cell volume shrinks (Supplementary Figs. 15 and 16). For Ba_1−*y*_Pb_*y*_TiO_3_, the enhancement of A-site stereochemical activity also leads to an obvious off-center shift of Ti^4+^ along the *c* axis, which in turn results in a significant contraction of the *a* axis, which compensates *c* axis elongation, so that the overall cell volume decreases. In contrast, the crystal structure for “114” oxides is highly strained in comparison with the flexible framework of perovskite, which can be deduced by the *c*/*a* value and bond valence sum (BVS) calculations^35,36^. The *c/a* value for CaBaZn_2_Ga_2_O_7_ (~ 1.605) is much smaller than the ideal closed-packing value (*c*/*a*_ideal_ = 1.633) and the BVS for Ca^2+^ in CaBaZn_2_Ga_2_O_7_ is estimated to be 2.48(1). All that suggests the fact that [Zn_2_Ga_2_O_7_]^4−^ anionic framework is highly strained along the *c* axis (or *c* axis is over-compressed), especially for the triangular layers because Ca^2+^ is seriously over-bonded (Fig. 4).

Structure strain is usually released through structure symmetry lowering and polyhedral distortion/rotation, which was observed indeed in numerous “114” oxides such as *Ln*BaFe_4_O_7_ (*Ln* = Y, Dy–Lu)^23,37^, *Ln*BaCo_4_O_7_ (*Ln* = Ca, Y, Tb–Lu)^38–40^, and *MA*Zn_2_Ga_2_O_7_ (*M* = Ca^2+^, Sr^2+^; *A* = Sr^2+^, Ba^2+^)^18^. Herein, the structure strain of CaBa_1−*x*_Pb_*x*_Zn_2_Ga_2_O_7_ is released without a significant distortion of the [Zn_2_Ga_2_O_7_]^4−^ framework, that is, the stereochemically active LP effect of Pb^2+^ helps the release of the structure strain. In detail, the LP active Pb^2+^ displaces from the center of the [Ba/PbO_12_] dodecahedron to form strong covalency bonds with O1, which attenuates the covalency of [Zn_2_Ga_2_O_7_]^4−^ anionic framework and drives displacement of O1 and O2 downwards along the *c* axis (Fig. 4b), resulting in a relief of uniaxial structure strain through a significant expansion of the *c* axis. Such a significant expansion for *c* axis further leads to an enhancement of the *c/a* value to 1.626 for CaPbZn_2_Ga_2_O_7_ and improvement of BVS value for Ca^2+^ in CaPbZn_2_Ga_2_O_7_ to 2.31(4), suggesting the anisotropic lattice-change and cell volume expansion is cooperative with the release of uniaxial structure strain along the *c* axis in CaBa_1−*x*_Pb_*x*_Zn_2_Ga_2_O_7_. Thus, the expansion of polar *c* axis for CaPbZn_2_Ga_2_O_7_ upon heating, which is different from the behavior of NTE materials, is also understandable. Finally, we can conclude that both the anisotropic chemical pressure induced by the highly stereochemical active LP electrons of Pb^2+^ and the uniaxial structure strain along *c* axis are responsible to the unprecedented cell volume expansion in CaBa_1−*x*_Pb_*x*_Zn_2_Ga_2_O_7_ solid solutions. In summary, substitution of Ba^2+^ in CaBaZn_2_Ga_2_O_7_ with the smaller but stereochemically active Pb^2+^ results in an unprecedented lattice volume expansion, which has not been observed in solid state chemistry to the best of our knowledge. Both reciprocal and direct space XRD and ND data analysis were utilized to decipher the origin of this unique phenomenon on the scale of both local and average structure of CaBa_1−*x*_Pb_*x*_Zn_2_Ga_2_O_7_. The results revealed that the LP electrons of Pb^2+^ are highly stereochemically active in this uniaxial greatly strained framework. Further DFT calculations revealed that the Pb^2+^ 6*s*6*p* orbitals are highly hybridized with O^2−^ 2*p* orbitals. The combined effect of these factors is the displacement of Pb^2+^ along the *c* axis, resulting in the release of structure strain associated with the axial cell expansion that is not compensated by contraction of the *ab*-plane and in turn produces the overall cell volume expansion of CaBa_1−*x*_Pb_*x*_Zn_2_Ga_2_O_7_. In general, our findings open new opportunities to use stereochemically active cations (Pb^2+^, Sn^2+^, Bi^3+^, etc.) to tune crystal structural strain, which has important role in ferroic materials.

## Methods

### Sample preparation

Polycrystalline samples of CaBa_1−*x*_Pb_*x*_Zn_2_Ga_2_O_7_ (0 ≤ *x* ≤ 1) and CaSr_0.5_Ba_0.5_Zn_2_Ga_2_O_7_ were prepared by conventional high temperature solid state reactions. Calcium carbonate (CaCO_3_, 99.99%), barium carbonate (BaCO_3_, 99.99%), strontium carbonate (SrCO_3_, 99.99%), lead oxide (PbO, 99.9%), zinc oxide (ZnO, 99.99%) and gallium oxide (Ga_2_O_3_, 99.99%) were used as starting materials. All the raw materials except for PbO were heated at 500 °C for 10 h before being weighted, in order to remove any adsorbed moisture. Stoichiometric raw materials were mixed and ground in an agate mortar and pre-heated at 800 °C for 10 h to decompose the carbonate. After this initial calcination, the resultant powder samples were re-ground thoroughly by hands and pressed into a pellet (*φ* = 13 mm). With increasing the doping content of Pb^2+^, the synthetic temperatures of CaBa_1−*x*_Pb_*x*_Zn_2_Ga_2_O_7_ decrease owing to the relative low melting point of PbO. The pellets were heated in the range of 860–1100 °C for 60 h with intermediate re-grindings. Moreover, for the preparation of CaBa_1−*x*_Pb_*x*_Zn_2_Ga_2_O_7_ (0.7 ≤ *x* ≤ 1), additional 10 mg PbO should be added to compensation the volatilization of PbO after every cycle of calcination. CaSr_0.5_Ba_0.5_Zn_2_Ga_2_O_7_ was prepared by heating raw materials at 1100 °C for 45 h with intermediate re-grindings.

### Structure characterizations

The phase purity of the samples can be ensured by powder XRD. XRD was performed on a PANalytical Empyrean powder diffractometer equipped with a PXIcel 1D detector. Room temperature constant ND data for CaBa_1−*x*_Pb_*x*_Zn_2_Ga_2_O_7_ (*x* = 0 and 0.5) (*λ* = 2.0775 Å) and CaPbZn_2_Ga_2_O_7_ (*λ* = 1.6215 Å) were collected at the BT-1 high-resolution ND diffractometer at the NIST Center for Neutron Research (NCNR) and ECHIDNA high-resolution powder diffractometer at the OPAL research facility (Lucas Heights, Australia)^41^, respectively. Combined Rietveld refinements on ND and X-ray data were performed using the TOPAS-Academic V6 software^42^.

Neutron total scattering experiments were performed at room temperature utilizing the nanoscale ordered materials diffractometer (NOMAD) at the spallation neutron source located at Oka Ridge National Laboratory. About 150 mg of each sample were loaded into a 2 mm diameter quartz capillary for measurements at room temperature with a collection time of ~ 2 h per sample. The PDF, G(r), was obtained through the Fourier transformation of S(Q) with Q value between 0.1 and 31.4 Å.

### DFT calculations

Theoretical study of CaPbZn_2_Ga_2_O_7_ was carried out using Vienna ab-initio simulation package (VASP)^43^. The projector augmented-wave method implemented in the VASP code was utilized to describe the interaction between the ionic cores and the valence electrons^44^. The generalized gradient approximation parameterized by Perdew, Burke, and Ernzerhof was employed to describe the exchange-correlation potential in the standard DFT calculations^45^. For single point energy and density of states, a cutoff energy of 500 eV for the plane-wave basis and 13 × 13 × 7 Monkhorst-Pack G-centered *k*-point meshes were employed.

## Supplementary information

Supplementary Information

Peer Review File

Supplementary Data 1

## Data Availability

All relevant data that support the results of this study are available from the corresponding author upon request.
