# Supplementary material for: Unprecedented lattice volume expansion on doping stereochemically active Pb2+ into uniaxially strained structure of CaBa1−xPbxZn2Ga2O7
Source: Nat Commun. 2020 Mar 11;11:1303. doi: 10.1038/s41467-020-14759-2 (PMC7066146; doi:10.1038/s41467-020-14759-2)
Supplement: Supplementary file 1 — Supplementary Information [file 41467_2020_14759_MOESM1_ESM.pdf]

Supplementary Information

Unprecedented lattice volume expansion on doping stereochemically active Pb^{2+} into uniaxially strained structure of $CaBa_{1-x}Pb_xZn_2Ga_2O_7$

Jiang et al.

Contents:

Supplementary Figures 1-16

Supplementary Tables 1-5

Supplementary Notes 1-3

Supplementary References

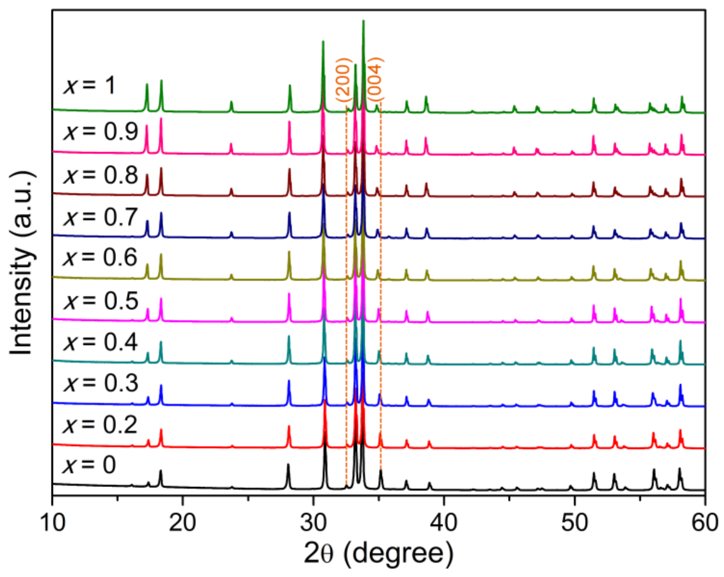

Supplementary Figure 1. Powder XRD patterns for $CaBa_{1-x}Pb_xZn_2Ga_2O_7$ (x = 0-1).

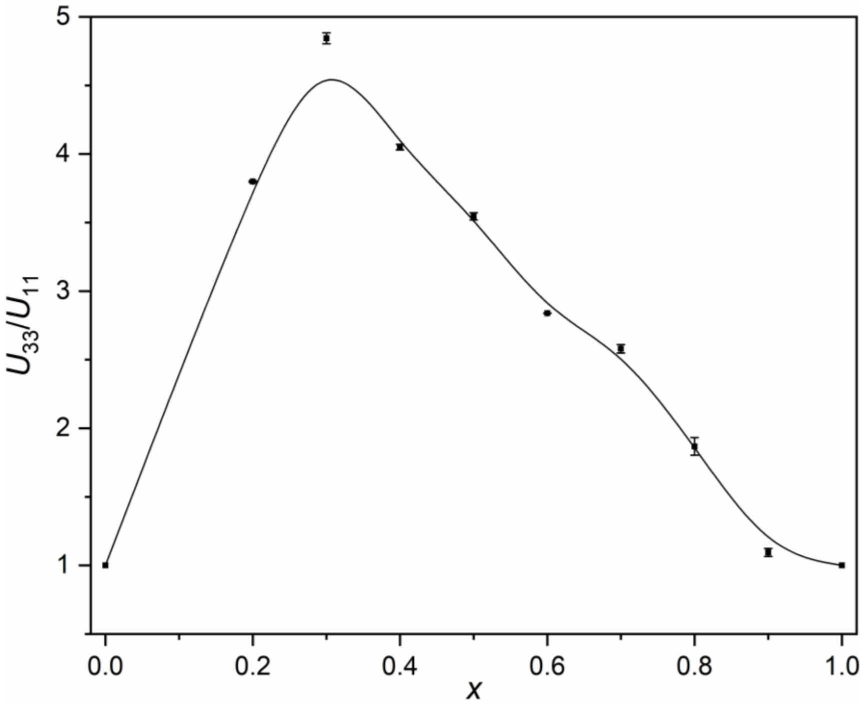

Supplementary Figure 2. Plots of U_{33}/U_{11} against Pb^{2+}-content in $CaBa_{1-x}Pb_xZn_2Ga_2O_7$.

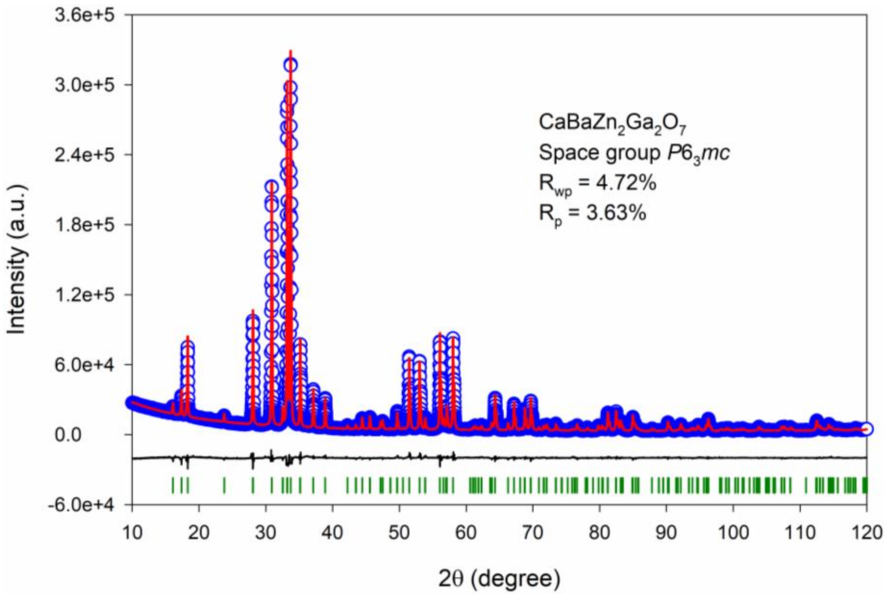
CaBaZn2Ga2O7
Space group P63mc
Rwp = 4.72%
Rp = 3.63%
Intensity (a.u.)
2θ (degree)
3.6e+5
3.0e+5
2.4e+5
1.8e+5
1.2e+5
6.0e+4
0.0
-6.0e+4
10 20 30 40 50 60 70 80 90 100 110 120

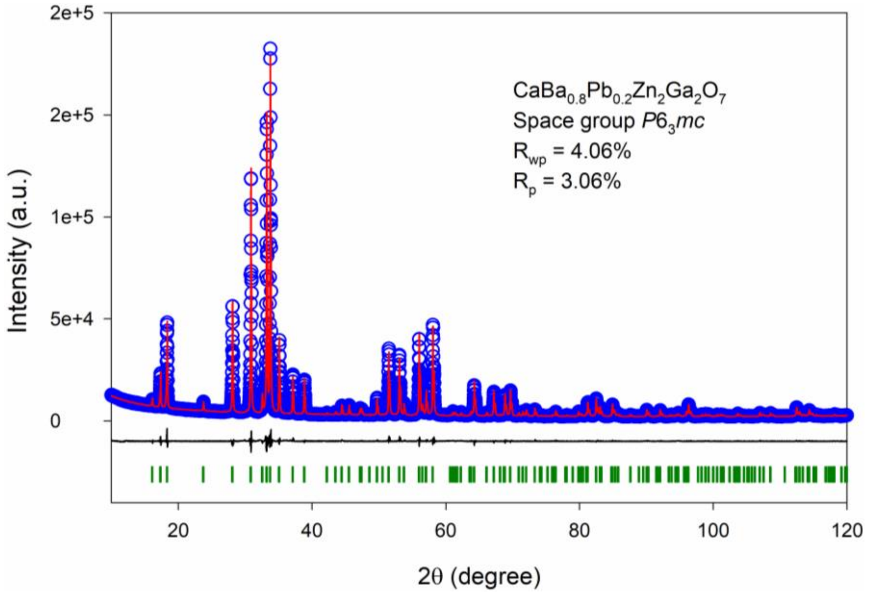
CaBa0.8Pb0.2Zn2Ga2O7
Space group P63mc
Rwp = 4.06%
Rp = 3.06%
Intensity (a.u.)
2θ (degree)
2e+5
2e+5
1e+5
5e+4
0
20 40 60 80 100 120

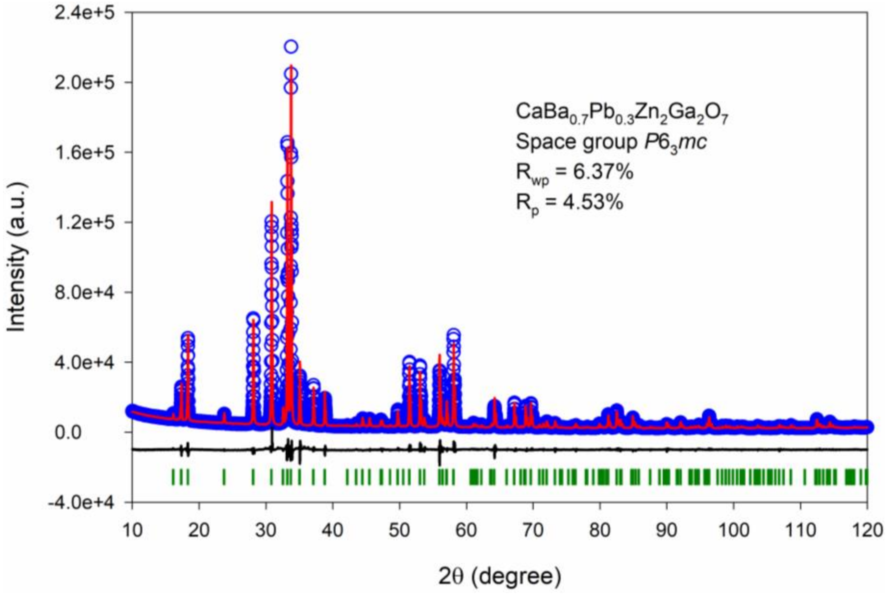
CaBa0.7Pb0.3Zn2Ga2O7
Space group P63mc
Rwp = 6.37%
Rp = 4.53%
Intensity (a.u.)
2θ (degree)
2.4e+5
2.0e+5
1.6e+5
1.2e+5
8.0e+4
4.0e+4
0.0
-4.0e+4
10 20 30 40 50 60 70 80 90 100 110 120

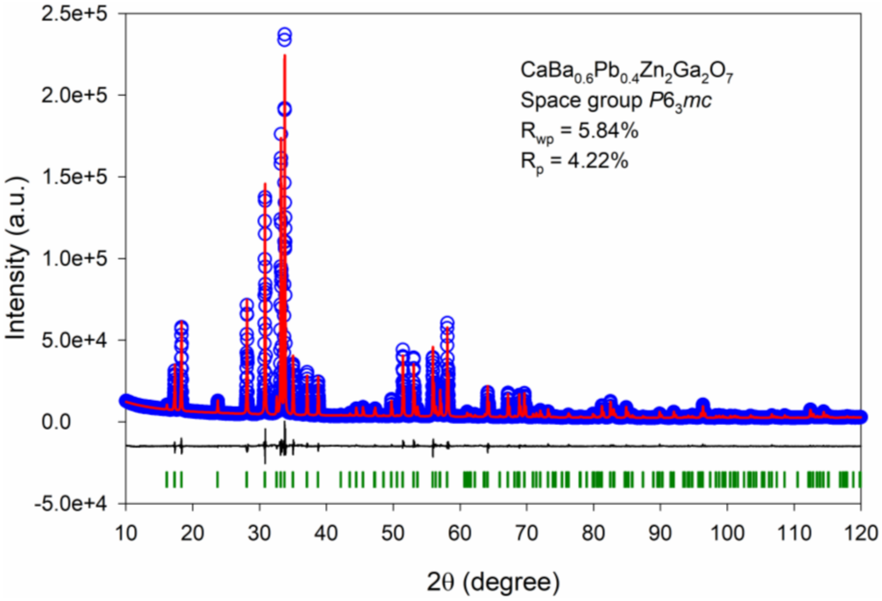

CaBa0.6Pb0.4Zn2Ga2O7
Space group P63mc
Rwp = 5.84%
Rp = 4.22%
Intensity (a.u.)
2.5e+5
2.0e+5
1.5e+5
1.0e+5
5.0e+4
0.0
-5.0e+4
10 20 30 40 50 60 70 80 90 100 110 120
2θ (degree)

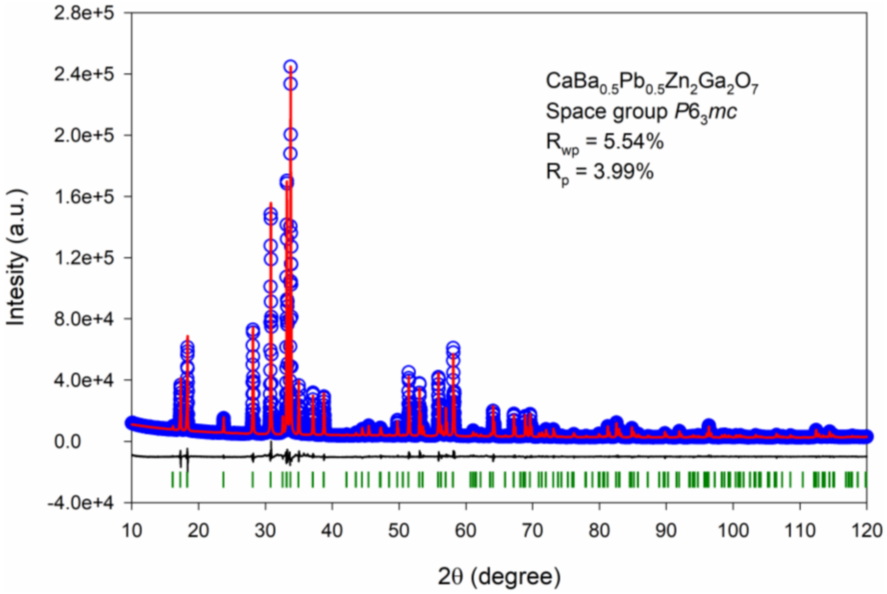

CaBa0.5Pb0.5Zn2Ga2O7
Space group P63mc
Rwp = 5.54%
Rp = 3.99%
Intesity (a.u.)
2.8e+5
2.4e+5
2.0e+5
1.6e+5
1.2e+5
8.0e+4
4.0e+4
0.0
-4.0e+4
10 20 30 40 50 60 70 80 90 100 110 120
2θ (degree)

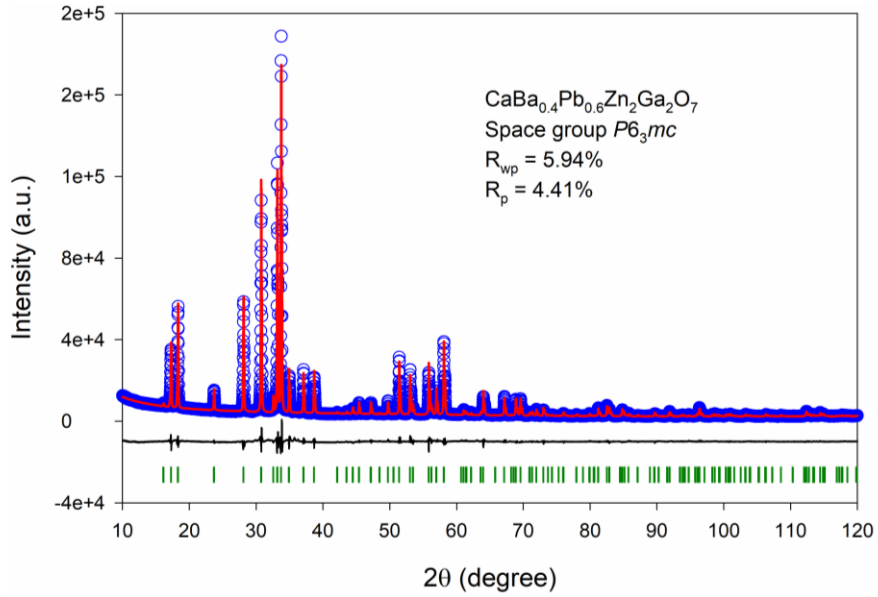

CaBa0.4Pb0.6Zn2Ga2O7
Space group P63mc
Rwp = 5.94%
Rp = 4.41%
Intensity (a.u.)
2e+5
2e+5
1e+5
8e+4
4e+4
0
-4e+4
10 20 30 40 50 60 70 80 90 100 110 120
2θ (degree)

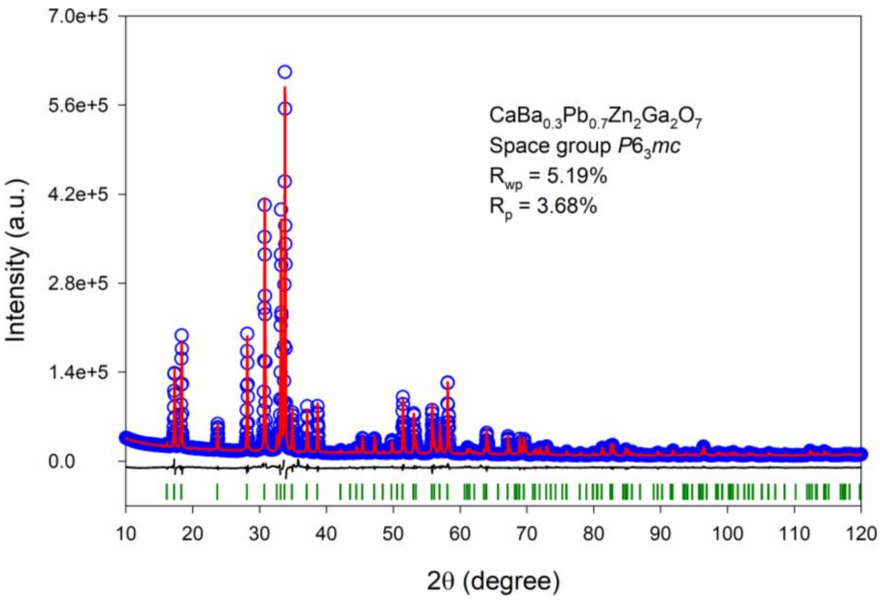
CaBa0.3Pb0.7Zn2Ga2O7
Space group P63mc
Rwp = 5.19%
Rp = 3.68%
Intensity (a.u.)
2θ (degree)

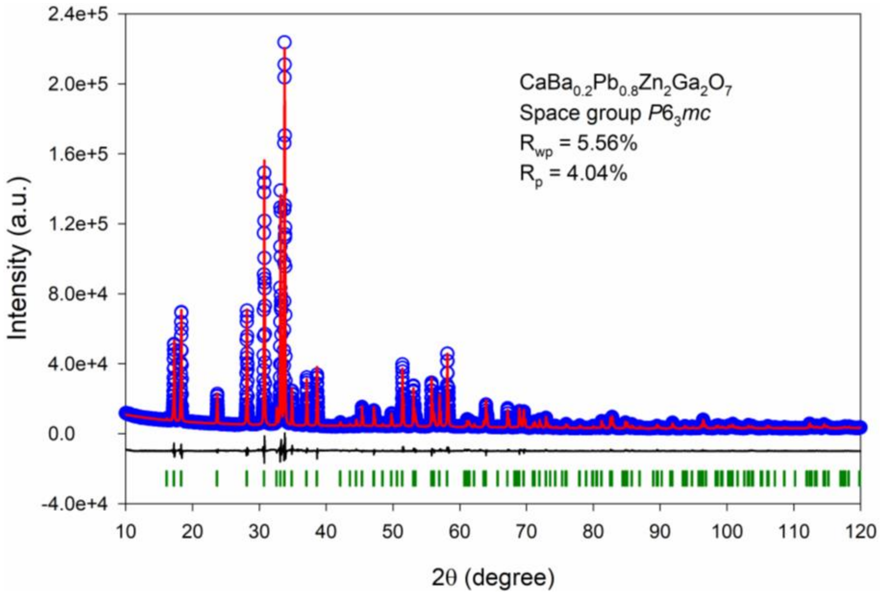
CaBa0.2Pb0.8Zn2Ga2O7
Space group P63mc
Rwp = 5.56%
Rp = 4.04%
Intensity (a.u.)
2θ (degree)

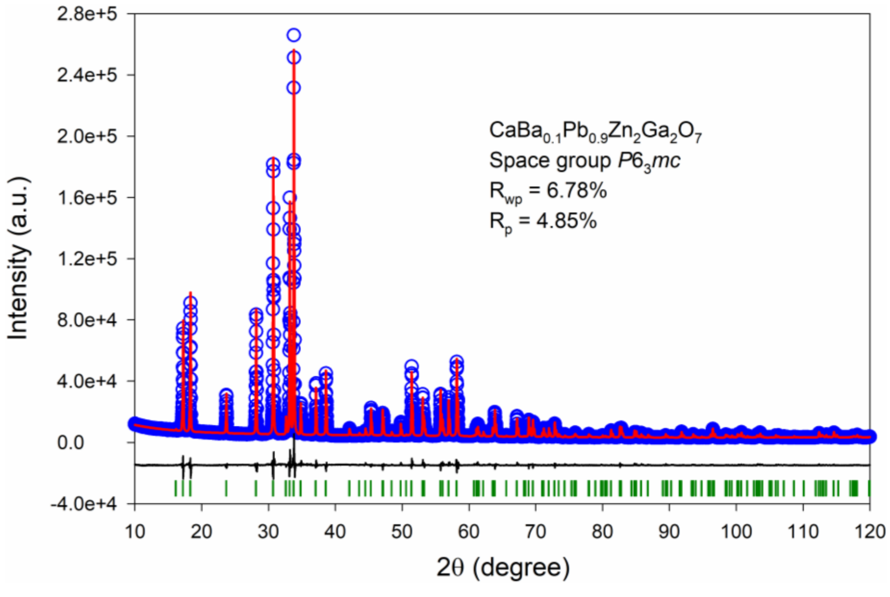
CaBa0.1Pb0.9Zn2Ga2O7
Space group P63mc
Rwp = 6.78%
Rp = 4.85%
Intensity (a.u.)
2θ (degree)

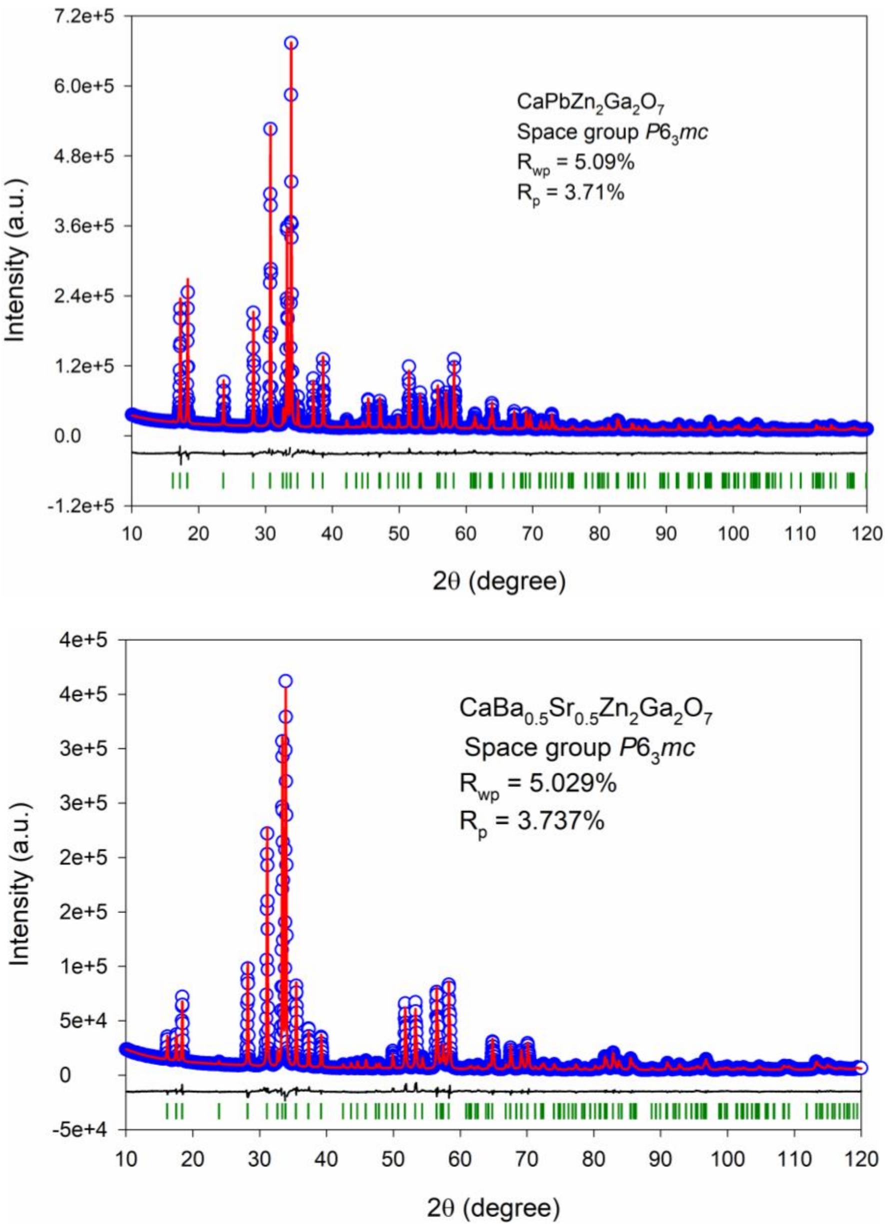

Supplementary Figure 3. Rietveld refinement plots on Cu K*α* XRD data for $CaBa_{1-x}Pb_xZn_2Ga_2O_7$ (x = 0, 0.2, 0.3, 0.4, 0.5, 0.6, 0.7, 0.8, 0.9, 1) and $CaBa_{0.5}Sr_{0.5}Zn_2Ga_2O_7$. The reliable factors are also given in right corner of the pattern. The blue symbol ○ represents the observed data and the solid red line is the calculated data. The difference curve and Bragg positions of the diffraction peaks are shown as black line and vertical bars at the bottom of the pattern, respectively.

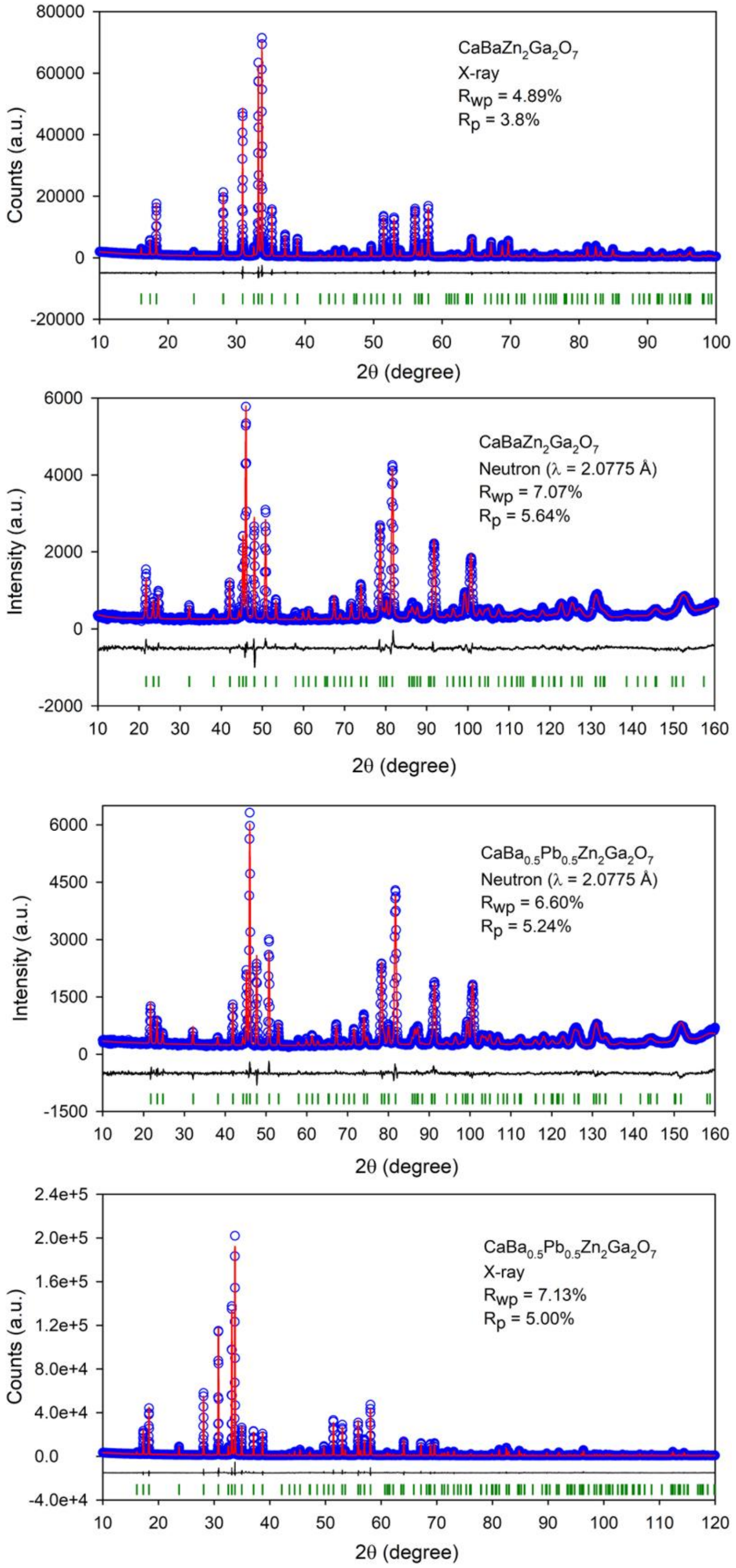

Supplementary Figure 4. Combined Rietveld refinement plots on Cu Kα1 XRD and ND data for $CaBaZn_2Ga_2O_7$ and $CaBa_{0.5}Pb_{0.5}Zn_2Ga_2O_7$ with space group $P6_3mc$.

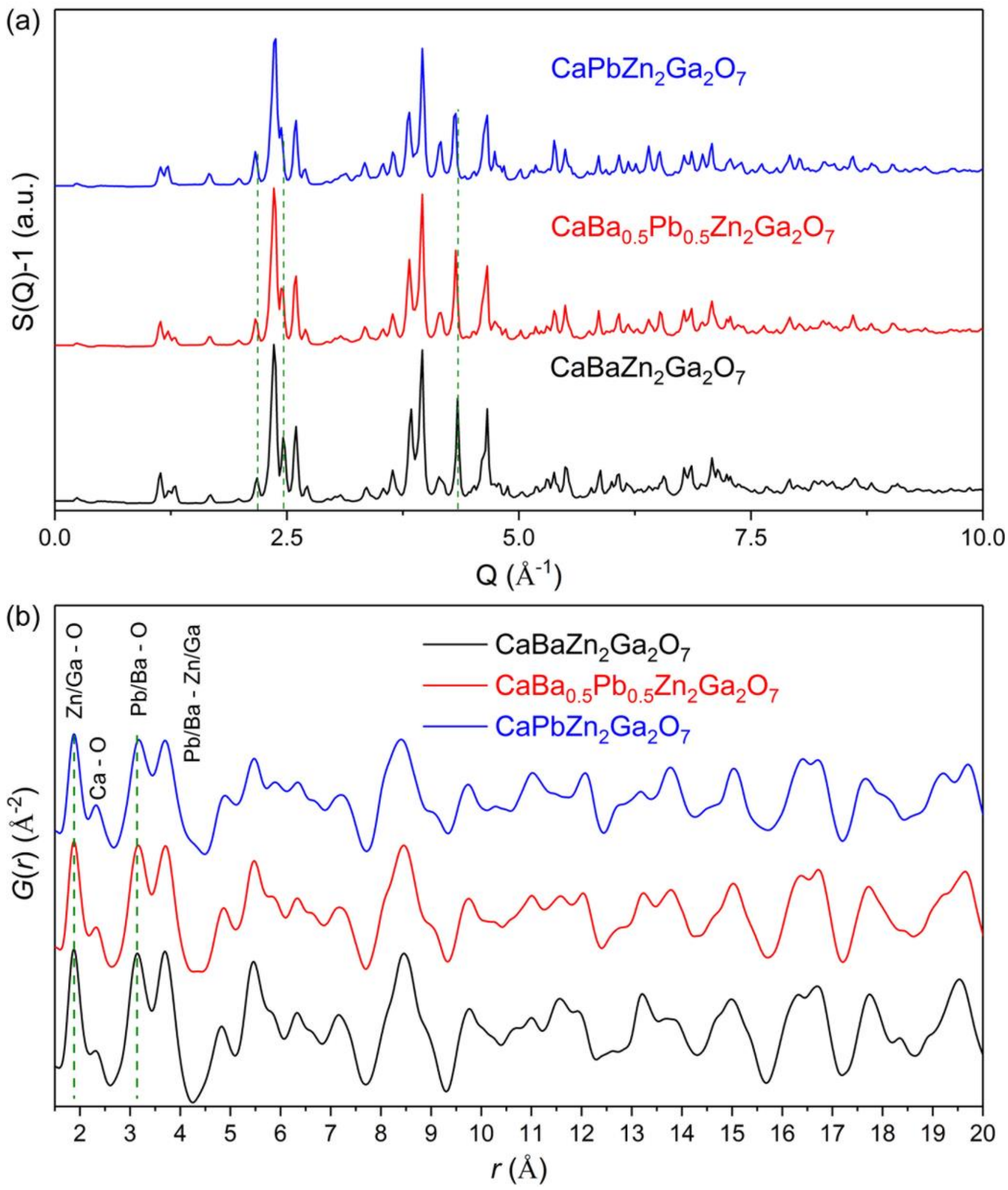

Supplementary Figure 5. (a) Normalized structure functions and (b) PDFs (pairs distribution functions, $G(r)$) for $Ca(Ba_{1-x}Pb_x)Zn_2Ga_2O_7$ (x = 0, 0.5 and 1).

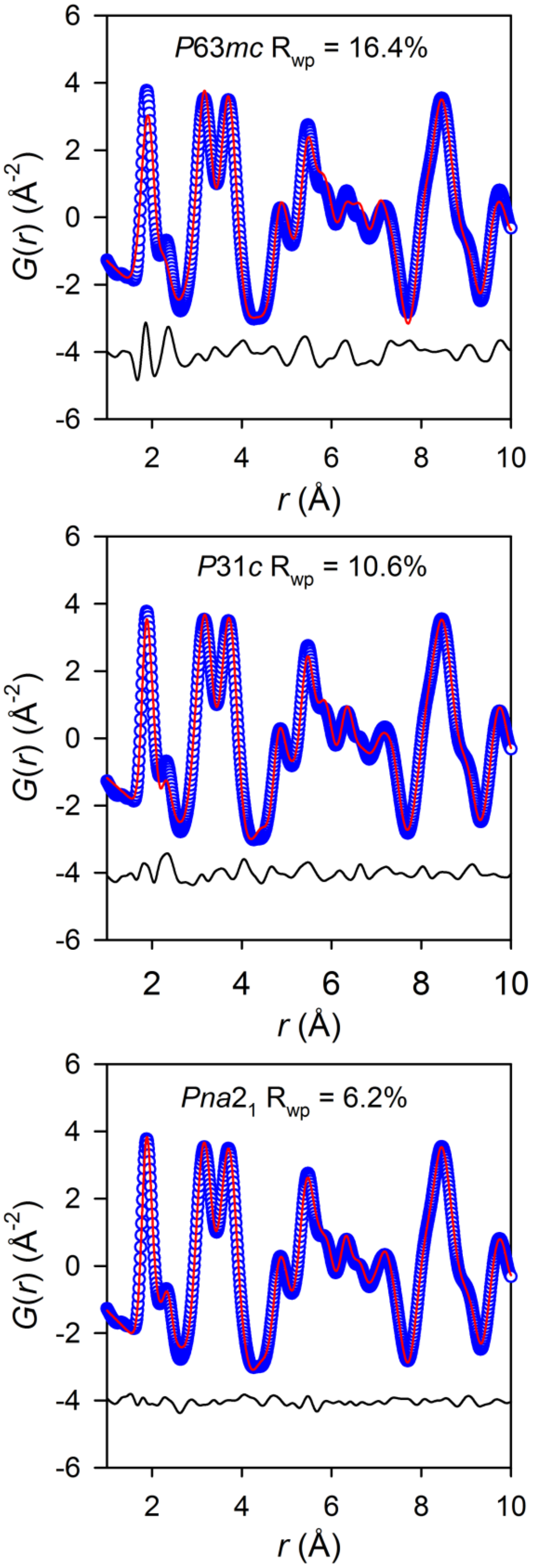

Supplementary Figure 6. Real space Rietveld refinement plots of neutron PDF data for $CaBa_{0.5}Pb_{0.5}Zn_2Ga_2O_7$ with *P*6$_3$*mc*, *P*31*c* and *Pna*2$_1$ models.

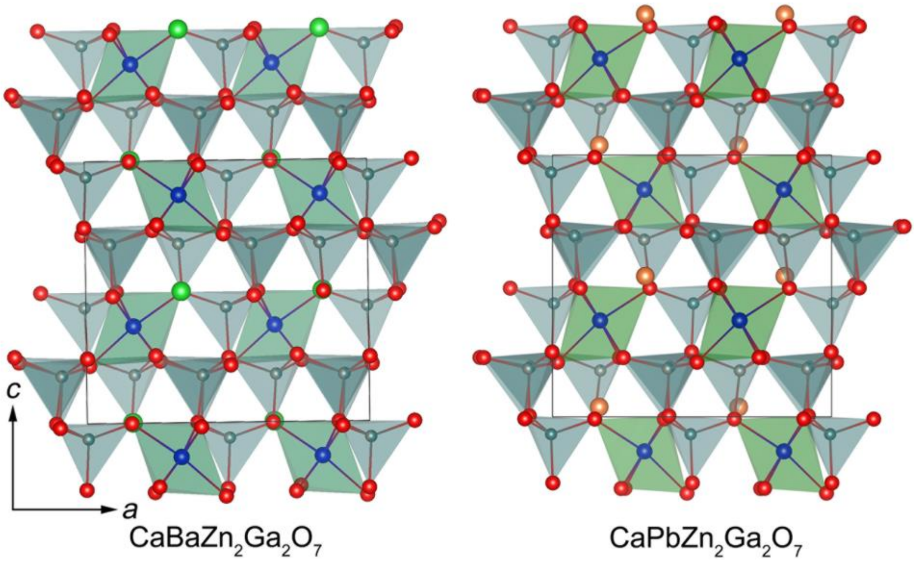

Supplementary Figure 7. Crystal structures for $CaBa_{1-x}Pb_xZn_2Ga_2O_7$ (x = 0 and 1) obtained from real space Rietveld refinements against neutron PDF data using space group $Pna2_1$.

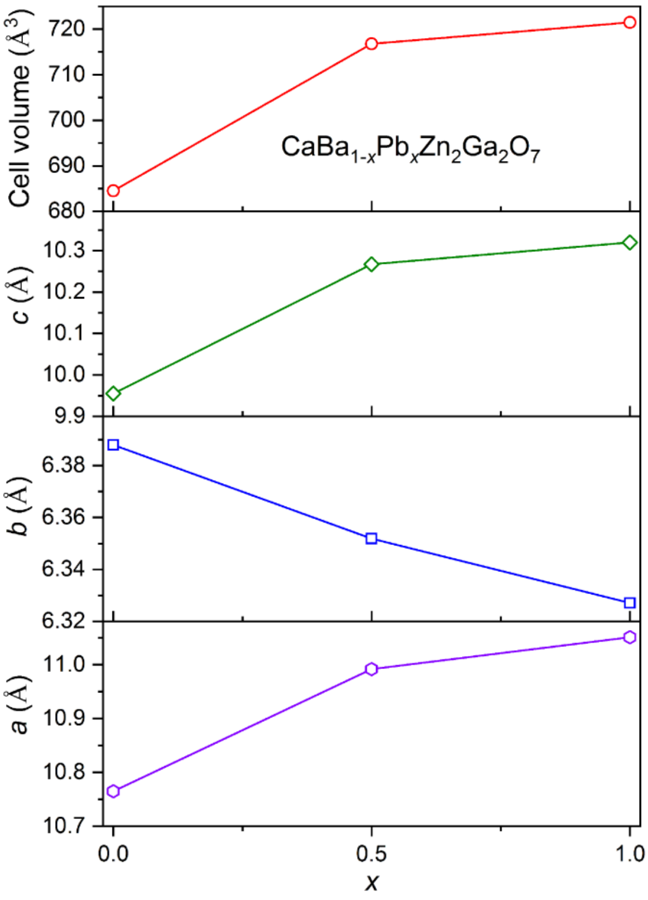

Supplementary Figure 8. Plots of lattice parameters extracted from PDF fitting for $CaBa_{1-x}Pb_xZn_2Ga_2O_7$ (x = 0, 0.5, and 1) with space group $Pna2_1$.

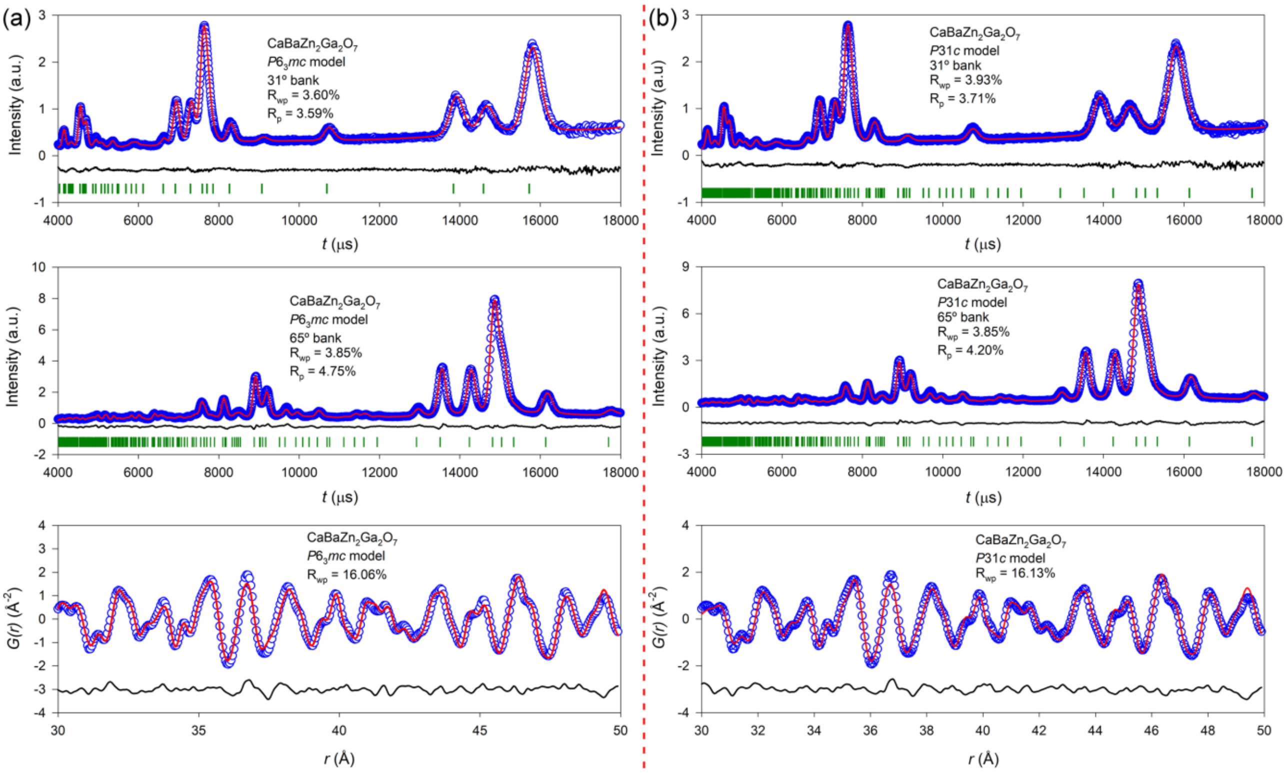

Supplementary Figure 9. Combined real and reciprocal space Rietveld refinements of time-of-flight ND data and PDF data for $CaBaZn_2Ga_2O_7$ with $P6_3mc$ (a) and $P31c$ (b) models. The reliable factors are also given in the patterns.

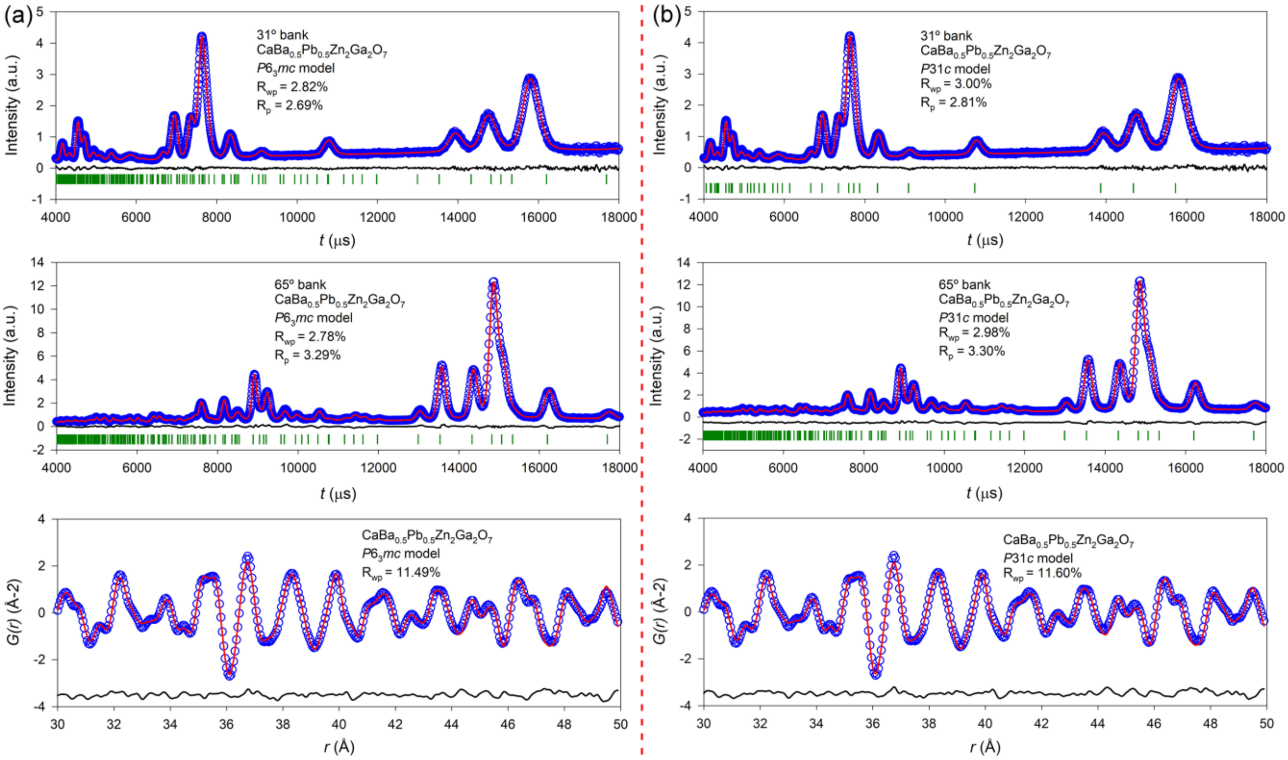

Supplementary Figure 10. Combined real and reciprocal space Rietveld refinements of time-of-

flight ND data and PDF data for $CaBa_{0.5}Pb_{0.5}Zn_2Ga_2O_7$ with $P6_3mc$ (a) and $P31c$ (b) models. The reliable factors are also given in the patterns.

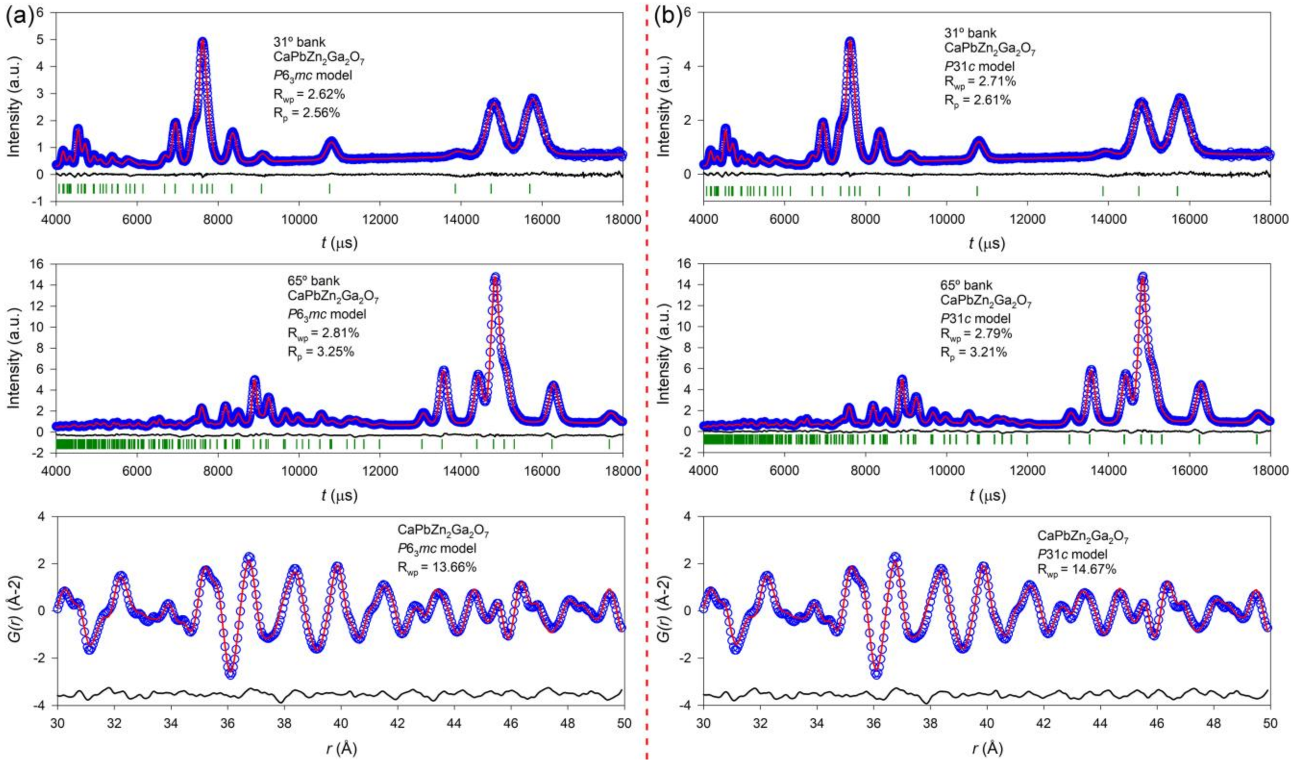

Supplementary Figure 11. Combined real and reciprocal space Rietveld refinements of time-of-flight ND data and PDF data for $CaPbZn_2Ga_2O_7$ with $P6_3mc$ (a) and $P31c$ (b) models. The reliable factors are also given in the patterns.

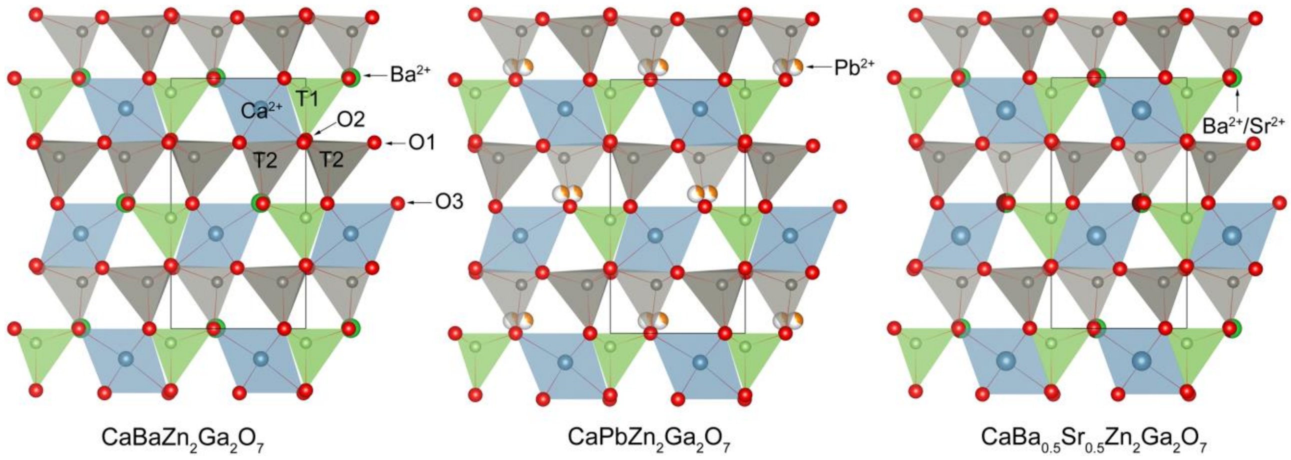

Supplementary Figure 12. Crystal structures for $CaBaZn_2Ga_2O_7$, $CaPbZn_2Ga_2O_7$ and $CaBa_{0.5}Sr_{0.5}Zn_2Ga_2O_7$ obtained from Rietveld refinement against Cu Kα XRD data with space

group $P6_3mc$. The significant structure change by Pb^{2+} doping is the deviation of Pb^{2+} from the "O3" layer along the *c*-axis due to the lone pair effect. In comparison with $CaBaZn_2Ga_2O_7$, no apparent structural change is observed for $CaBa_{0.5}Sr_{0.5}Zn_2Ga_2O_7$.

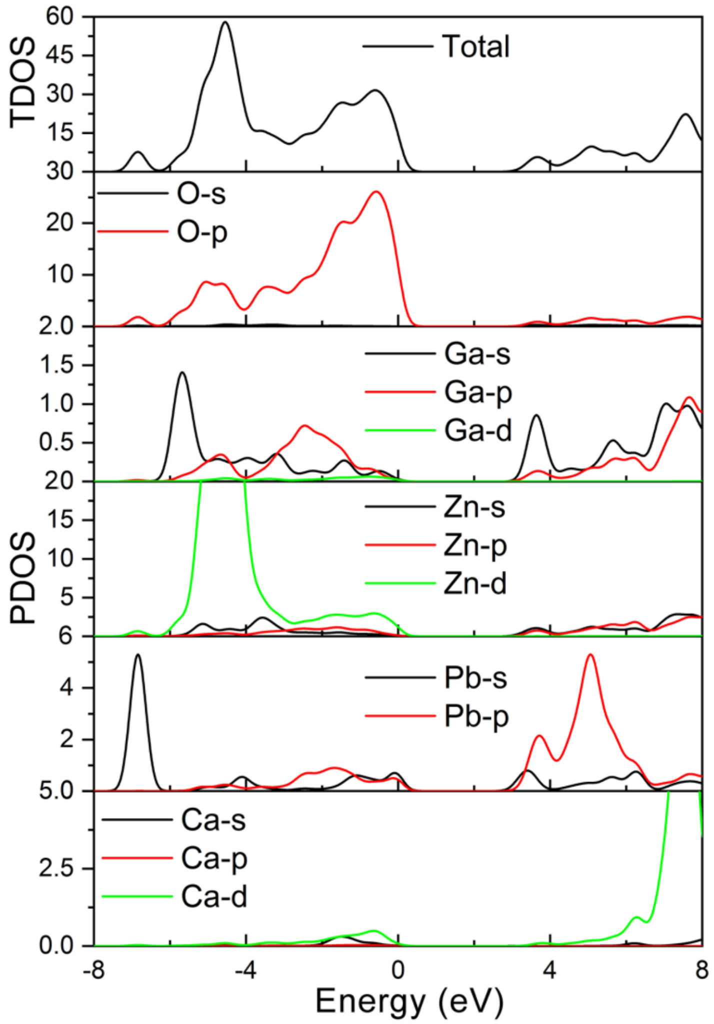

Supplementary Figure 13. Calculated DOS patterns of $CaPbZn_2Ga_2O_7$. The 6*s* and 6*p* orbitals of Pb^{2+} contribute to both the top of valence band and the bottom of conduction band, which suggests the orbital hybridization between Pb^{2+}(6*s*6*p*) and O^{2-}(2*p*).

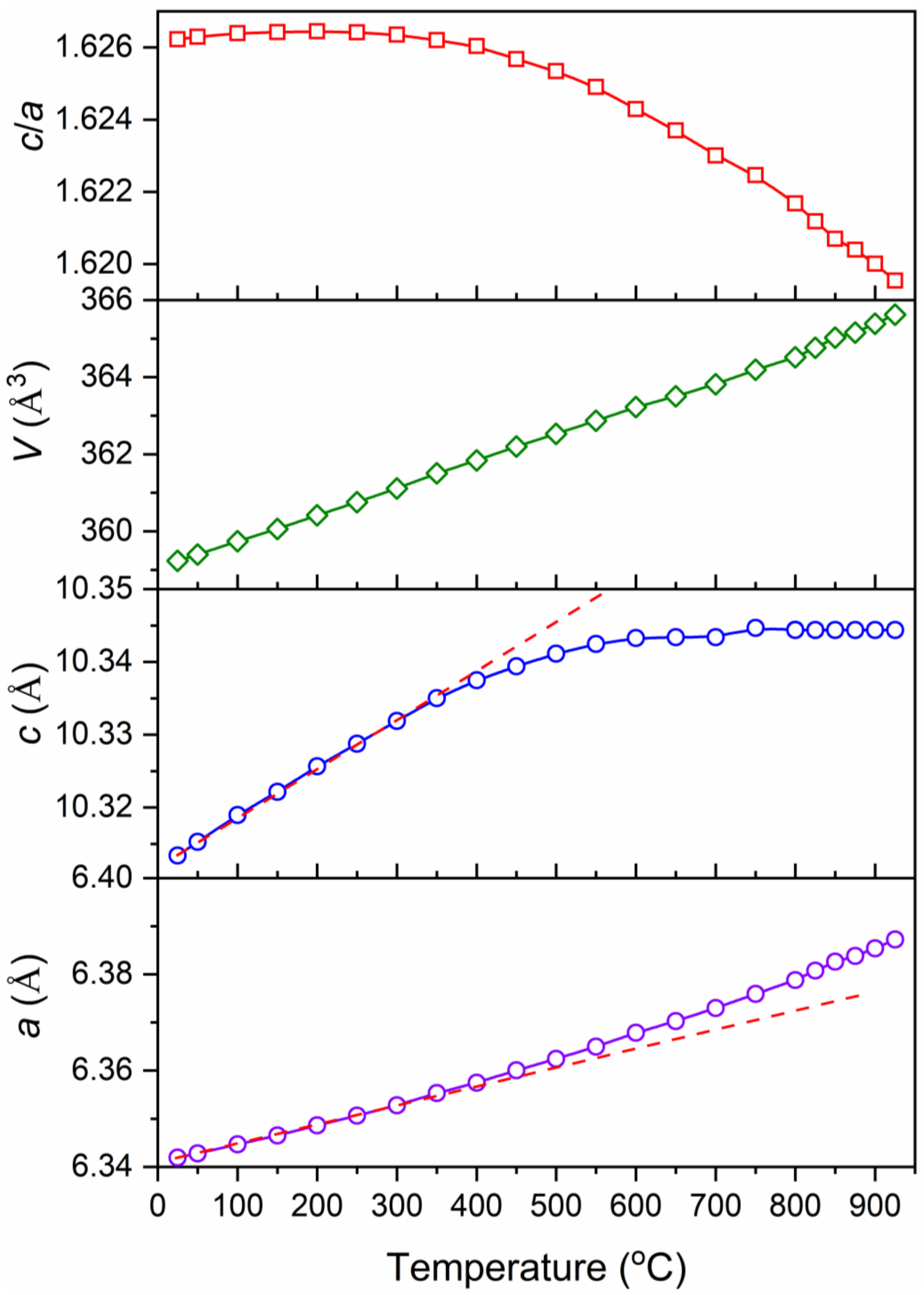

Supplementary Figure 14. Plots of lattice parameters for $CaPbZn_2Ga_2O_7$ against temperatures by in-situ high temperature X-ray diffraction.

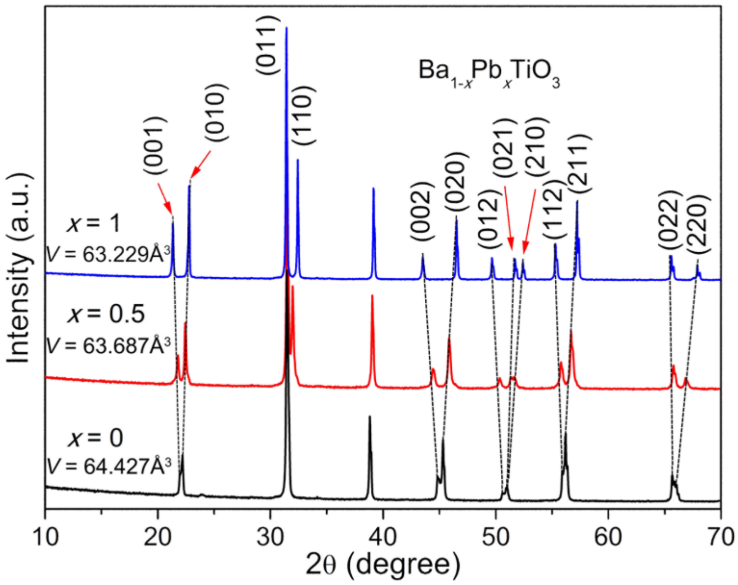

Supplementary Figure 15. XRD patterns for $Ba_{1-x}Pb_xTiO_3$ (x = 0, 0.5, and 1).

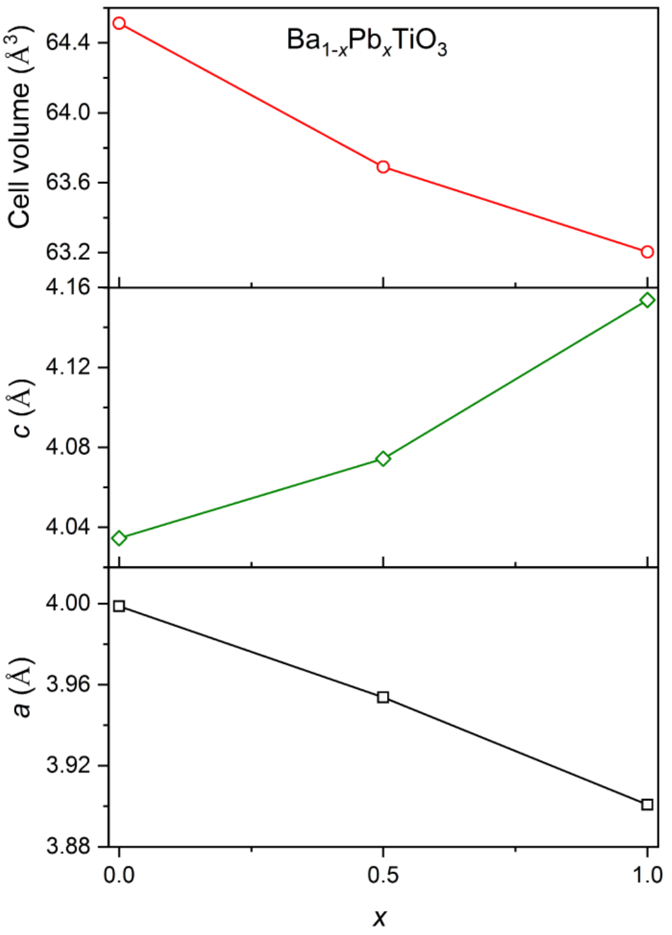

Supplementary Figure 16. Lattice parameters for $Ba_{1-x}Pb_xTiO_3$ (x = 0, 0.5, and 1) extracted from Le-bail fitting.

Supplementary Table 1. The comparison of cell volumes for isostructural compounds containing Ba^{2+} and Pb^{2+}.

Compound	Structure type	Cell volume (Å^3)	Volume of performula (Å^3)
$BaWO_4$[2]	Scheelite	361.85	90.25
$PbWO_4$[3]	Scheelite	357.30	89.325
$BaGa_2O_4$[4]	Tridymite	866.58 (Z = 8)	108.323
$PbGa_2O_4$[5]	Tridymite	421.83 (Z = 4)	105.458
$BaFe_{12}O_{19}$[6]	Hexagonal ferrite	696.20	348.10
$PbFe_{12}O_{19}$[6]	Hexagonal ferrite	694.90	347.45
$BaBiNb_5O_{15}$[7]	Tungsten-bronze	616.31 (Z = 2)	308.155
$PbBiNb_5O_{15}$[8]	Tungsten-bronze	2420.08 (Z = 8)	302.51
$BaNb_2O_6$[9]	Tungsten-bronze	248.91 (Z = 2)	124.455
$PbNb_2O_6$[10]	Tungsten-bronze	4905.25 (Z = 40)	122.631
$BaTiO_3$[a]	Perovskite	64.427	64.427
$PbTiO_3$[a]	Perovskite	63.229	63.229
$Ba_3P_2O_8$[11]	Palmierite	571.29 (Z = 3)	190.43
$Pb_3P_2O_8$[12]	Palmierite	692.78 (Z = 4)	174.195

[a] See detail in Supplementary Figs. S15 and S16.

Supplementary Table 2. Atomic coordinates, site occupancies and thermal displacement factors for $CaBa_{1-x}Pb_xZn_2Ga_2O_7$ (x = 0-1.0) and $CaBa_{0.5}Sr_{0.5}Zn_2Ga_2O_7$ obtained from Rietveld refinement against XRD data with space group $P6_3mc$.[a]

x = 0	x	y	z	Occ.	$U_{iso.}$(Å^2)
Ca	2/3	1/3	0.8798(8)	1	0.0079(9)
Ba	2/3	1/3	0.5024(7)	1	0.0242(8)
T1	0	0	0.4485(7)	1	0.0145(7)
T2	0.17192(8)	0.82808(1)	0.6909(6)	1	0.0121(7)
O1	0.5082(7)	0.4918(7)	0.7474(7)	1	0.034(2)
O2	0	0	0.2615(9)	1	0.023(2)
O3	0.1592(5)	0.8408(5)	0.5	1	0.039(2)

$x = 0.2$	x	y	z	Occ.	$U_{11} = U_{22} = U_{12}$ (Å^2)	U_{33} (Å^2)
Ca	2/3	1/3	0.8744(8)	1	0.0036(9)	
Ba/Pb	2/3	1/3	0.5062(7)	0.8/0.2	0.0179(5)	0.068(2)
T1	0	0	0.4400(7)	1	0.0106(8)	
T2	0.17143(8)	0.82857(1)	0.6837(6)	1	0.0075(7)	
O1	0.4989(9)	0.5011(9)	0.7393(8)	1	0.033(2)	
O2	0	0	0.249(1)	1	0.012(2)	
O3	0.1559(5)	0.8441(5)	0.5	1	0.037(2)	
$x = 0.3$	x	y	z	Occ.	$U_{11} = U_{22} = U_{12}$ (Å^2)	U_{33} (Å^2)
Ca	2/3	1/3	0.8850(9)	1	0.008(1)	
Ba/Pb	2/3	1/3	0.5204(9)	0.7/0.3	0.0223(8)	0.108(3)
T1	0	0	0.4510(9)	1	0.013(1)	
T2	0.1721(1)	0.8279(1)	0.6914(7)	1	0.0090(9)	
O1	0.502(1)	0.498(1)	0.747(1)	1	0.029(4)	
O2	0	0	0.261(2)	1	0.028(5)	
O3	0.1543(8)	0.8457(8)	0.5	1	0.029(3)	
$x = 0.4$	x	y	z	Occ.	$U_{11} = U_{22} = U_{12}$ (Å^2)	U_{33} (Å^2)
Ca	2/3	1/3	0.8828(7)	1	0.004(1)	
Ba/Pb	2/3	1/3	0.5225(8)	0.6/0.4	0.0279(6)	0.113(3)
T1	0	0	0.4482(8)(4)	1	0.012(1)	
T2	0.1719(1)	0.8281(1)	0.6900(6)	1	0.0083(8)	
O1	0.5033(9)	0.4967(9)	0.7450(9)	1	0.028(3)	
O2	0	0	0.262(2)	1	0.039(5)	
O3	0.1534(7)	0.8466(7)	0.5	1	0.027(3)	
$x = 0.5$	x	y	z	Occ.	$U_{11} = U_{22} = U_{12}$ (Å^2)	U_{33} (Å^2)
Ca	2/3	1/3	0.8820(7)	1	0.004(1)	
Ba/Pb	2/3	1/3	0.5264(7)	0.5/0.5	0.0330(6)	0.117(3)
T1	0	0	0.4475(8)	1	0.0106(9)	
T2	0.1720(1)	0.8280(1)	0.6695(6)	1	0.0078(8)	
O1	0.5020(9)	0.4980(9)	0.7435(9)	1	0.026(3)	
O2	0	0	0.259(1)	1	0.018(4)	
O3	0.1550(7)	0.8450(7)	0.5	1	0.022(3)	
$x = 0.6$	x	y	z	Occ.	$U_{11} = U_{22} = U_{12}$ (Å^2)	U_{33} (Å^2)
Ca	2/3	1/3	0.8779(8)	1	0.011(1)	

Ba/Pb	2/3	1/3	0.5285(9)	0.4/0.6	0.0384(7)	0.109(2)
T1	0	0	0.4425(9)	1	0.011(1)	
T2	0.1718(2)	0.8282(1)	0.6864(7)	1	0.0078(9)	
O1	0.5022(9)	0.4978(9)	0.7334(9)	1	0.029(3)	
O2	0	0	0.220(1)	1	0.027(5)	
O3	0.1507(8)	0.8493(8)	0.5	1	0.027(3)	
x = 0.7	*x*	*y*	*z*	Occ.	$U_{11} = U_{22} = U_{12}$ (Å^2)	U_{33} (Å^2)
Ca	2/3	1/3	0.883(1)	1	0.014(3)	
Ba/Pb	2/3	1/3	0.5382(9)	0.3/0.7	0.038(2)	0.098(4)
T1	0	0	0.448(1)	1	0.009(2)	
T2	0.1718(2)	0.8282(2)	0.690(1)	1	0.010(2)	
O1	0.504(1)	0.496(1)	0.735(2)	1	0.020(3)	
O2	0	0	0.260(2)	1	0.020(3)	
O3	0.147(1)	0.853(1)	0.5	1	0.020(3)	
x = 0.8	*x*	*y*	*z*	Occ.	$U_{11} = U_{22} = U_{12}$ (Å^2)	U_{33} (Å^2)
Ca	2/3	1/3	0.8848(8)	1	0.018(1)	
Ba/Pb	2/3	1/3	0.5429(7)	0.2/0.8	0.0455(8)	0.085(2)
T1	0	0	0.4483(8)	1	0.0059(9)	
T2	0.1710(3)	0.8290(3)	0.6915(6)	1	0.0055(8)	
O1	0.5030(9)	0.4970(9)	0.740(1)	1	0.022(1)	
O2	0	0	0.262(1)	1	0.022(1)	
O3	0.1491(9)	0.8509(9)	0.5	1	0.022(1)	
x = 0.9	*x*	*y*	*z*	Occ.	$U_{11} = U_{22} = U_{12}$ (Å^2)	U_{33} (Å^2)
Ca	2/3	1/3	0.8847(8)	1	0.022(2)	
Ba/Pb	2/3	1/3	0.5466(7)	0.1/0.9	0.0475(8)	0.052(2)
T1	0	0	0.4485(8)	1	0.003(1)	
T2	0.1716(2)	0.8284(2)	0.6904(6)	1	0.004(1)	
O1	0.5065(8)	0.4935(8)	0.7341(9)	1	0.023(2)	
O2	0	0	0.259(1)	1	0.023(2)	
O3	0.1525(9)	0.8475(9)	0.5	1	0.023(2)	
x = 1	*x*	*y*	*z*	Occ.	$U_{iso.}$ (Å^2)	
Ca	2/3	1/3	0.8852(9)	1	0.016(2)	
Pb	0.6346(4)	0.3654(4)	0.5494(9)	1/3	0.0327(8)	
T1	0	0	0.447(1)	1	0.003(1)	
T2	0.1710(2)	0.8289(2)	0.6917(8)	1	0.005(1)	
O1	0.502(1)	0.498(2)	0.740(1)	1	0.015(3)	

O2	0	0	0.257(2)	1	0.015(3)
O3	0.149(1)	0.851(1)	0.5	1	0.015(3)
$Ba_{0.5}Sr_{0.5}$	*x*	*y*	*z*	Occ.	$U_{iso.}$ (Å^2)
Ca	2/3	1/3	0.8698(4)	1	0.017(1)
Ba/Sr	2/3	1/3	0.5	0.53(1)/0.47(1)	0.0114(8)
T1	0	0	0.4403(5)	1	0.0127(8)
T2	0.1713(1)	0.8287(3)	0.6845(3)	1	0.015(6)
O1	0.493(1)	0.507(1)	0.7371(8)	1	0.046(4)
O2	0	0	0.249(1)	1	0.037(4)
O3	0.1580(8)	0.8420(1)	0.501(1)	1	0.048(4)

[a] U_{ij} indicates anisotropic thermal displacement parameters. U_{13} and U_{23} are zero.

Supplementary Table 3. Selected bond distances (Å) of $CaBa_{1-x}Pb_xZn_2Ga_2O_7$ and $CaBa_{0.5}Sr_{0.5}Zn_2Ga_2O_7$ obtained from Rietveld refinement against XRD data with space group $P6_3mc$.[a]

Bonds	x = 0	x = 0.2	x = 0.3	x = 0.4	x = 0.5	x = 0.6
Ca-O1×3	2.232(9)	2.31(1)	2.23(1)	2.29(1)	2.30(1)	2.34(1)
Ca-O3×3	2.276(7)	2.337(6)	2.295(9)	2.315(8)	2.306(8)	2.369(9)
Ba/Pb-O1×3	3.01(1)	3.02(1)	2.94(1)	2.90(1)	2.87(1)	2.78(1)
Ba/Pb -O3×6	3.1779(3)	3.1788(3)	3.1853(8)	3.1865(7)	3.1888(8)	3.193(1)
Ba/Pb-O1×3	3.27(1)	3.28(1)	3.36(1)	3.40(1)	3.45(1)	3.56(1)
T1-O3×3	1.829(6)	1.822(6)	1.770(9)	1.769(8)	1.787(8)	1.759(9)
T1-O2	1.91(1)	1.95(1)	1.95(2)	1.91(2)	1.94(2)	1.92(2)
T2-O1×2	1.943(5)	1.889(6)	1.903(8)	1.910(7)	1.899(7)	1.881(7)
T2-O3	1.953(6)	1.887(9)	1.969(7)	1.956(6)	1.955(6)	1.929(7)
T2-O2	2.024(5)	2.002(5)	2.021(1)	2.031(7)	2.021(6)	2.019(6)
Bonds	x = 0.7	x = 0.8	x = 0.9	x = 1.0	$CaBa_{0.5}Sr_{0.5}Zn_2Ga_2O_7$	
Ca-O1×3	2.35(2)	2.34(1)	2.32(1)	2.34(1)		2.33(1)
Ca-O3×3	2.38(1)	2.347(9)	2.35(1)	2.35(1)		2.34(1)
Ba/Pb-O1×3	2.70(2)	2.71(1)	2.61(1)	2.69(2)		3.07(1)

Ba/Pb -O3×6	3.206(2)	3.209(1)	3.212(1)	3.227(9)	3.1667(3)
Ba/Pb-O1×3	3.64(2)	3.64(1)	3.74(1)	3.708(6)	3.19(1)
T1-O3×3	1.70(1)	1.72(1)	1.76(1)	1.73(1)	1.83(9)
T1-O2	1.93(2)	1.92(2)	1.96(1)	1.96(2)	1.94(1)
T2-O1×2	1.89(1)	1.891(7)	1.897(6)	1.886(8)	1.841(7)
T2-O3	1.98(1)	1.987(6)	1.974(6)	1.991(9)	1.87(1)
T2-O2	2.02(8)	2.019(6)	2.012(5)	1.996(7)	1.989(5)

[a] For $CaPbZn_2Ga_2O_7$, Pb-O3 and Pb-O1 represent the average < Pb-O3> and <Pb-O1> bond lengths.

Supplementary Table 4. Atomic coordinates, isotropic thermal factors for $CaBa_{1-x}Pb_xZn_2Ga_2O_7$ (x = 0, 0.5, and 1.0) obtained from Rietveld refinement with space group $P6_3mc$ against constant wavelength ND and Cu Kα1 XRD data.

x = 0	x	y	z	Occ.	$U_{iso.}$(Å^2)	
Ca	2/3	1/3	0.881(2)	1	0.0038(6)	
Ba	2/3	1/3	0.5028(4)	1	0.0173(3)	
Zn1/Ga1	0	0	0.4436(4)	0.21(3)/0.79(3)	0.0054(4)	
Zn2/Ga2	0.17154(7)	0.82846(4)	0.6865(3)	0.60(1)/0.40(1)	0.0082(3)	
O1	0.5010(4)	0.4990(4)	0.7435(5)	1	0.0404(9)	
O2	0	0	0.2531(7)	1	0.011(1)	
O3	0.1566(3)	0.8434(3)	0.5	1	0.0433(6)	
x = 0.5	x	y	z	Occ.	$U_{11} = U_{22} = U_{12}$ (Å^2)	U_{33} (Å^2)
Ca	2/3	1/3	0.8790(4)	1	0.0020(2)	
Ba/Pb	2/3	1/3	0.5237(5)	0.578(6)/0.422(6)	0.0320(4)	0.112(2)
Zn1/Ga1	0	0	0.3991(5)	0.21(5)/0.79(5)	0.0087(5)	
Zn2/Ga2	0.17202(9)	0.82798(9)	0.6876(4)	0.60(2)/0.4(2)	0.0078(3)	
O1	0.5009(6)	0.4991(6)	0.6932(7)	1	0.035(2)	

O2	0	0	0.2128(8)	1	0.0078(3)
O3	0.1542(4)	0.8458(4)	0.5	1	0.0457(5)
x = 1	*x*	*y*	*z*	Occ.	$U_{iso.}$ (Å^2)
Ca	2/3	1/3	0.8785(3)	1	0.0145(6)
Pb	0.6334(1)	0.3666(1)	0.5464(3)	1/3	0.0274(5)
Zn1/Ga1	0	0	0.4433(3)	0.21(3)/0.79(3)	0.0066(4)
Zn2/Ga2	0.17105(8)	0.82895(8)	0.6874(2)	0.60(2)/0.4(2)	0.009(2)
O1	0.5073(2)	0.4927(2)	0.7288(7)	1	0.0214(5)
O2	0	0	0.2547(7)	1	0.0093(7)
O3	0.1533(2)	0.8467(2)	0.5	1	0.0379(3)

Supplementary Table 5. Selected bond lengths (Å) for $CaBa_{1-x}Pb_xZn_2Ga_2O_7$ (*x* = 0, 0.5, and 1.0) obtained from combined Rietveld refinement with the $P6_3mc$ model against constant wavelength ND and Cu Kα1 XRD data.

Bonds	Length (Å)	Bonds	Length (Å)	Bonds	Length (Å)
		$CaBaZn_2Ga_2O_7$			
Ca-O1×3	2.257(6)	Ba-O3×6	3.1798(1)	Zn1/Ga1-O2	1.924(9)
Ca-O3×3	2.332(3)	Ba-O1×3	3.225(6)	Zn2/Ga2-O1×2	1.906(3)
Ba-O1×3	3.059(5)	Zn1/Ga1-O3×3	1.818(3)	Zn2/Ga2-O3×1	1.909(6)
				Zn2/Ga2-O2×1	2.013(3)
		$CaBa_{0.5}Pb_{0.5}Zn_2Ga_2O_7$			
Ca-O1×3	2.305(7)	Ba/Pb -O3×6	3.1962(4)	Zn1/Ga1-O2	1.91(1)
Ca-O3×3	2.328(4)	Ba/Pb -O1×3	3.442(8)	Zn2/Ga2-O1×2	1.893(4)
Ba/Pb-O1×3	2.875(8)	Zn1/Ga1-O3×3	1.787(4)	Zn2/Ga2-O3×1	1.935(4)
				Zn2/Ga2-O2×1	2.026(4)
		$CaPbZn_2Ga_2O_7$			

Ca-O1×3	2.336(5)	Pb-O3×2	3.2140(5)	Zn1/Ga1-O2	1.947(8)
Ca-O3×3	2.340(3)	Pb-O3×2	3.535(1)	Zn2/Ga2-O1×2	1.898(2)
Pb-O1	2.337(6)	Pb-O1	3.621(7)	Zn2/Ga2-O2	1.942(3)
Pb-O1×2	2.716(5)	Pb-O1×2	3.900(6)	Zn2/Ga2-O3	2.002(3)
Pb-O3×2	2.913(1)	Zn1/Ga1-O3×3	1.783(3)		

Supplementary Note 1. Characterization.

The phase purity of the samples was investigated by powder X-ray diffraction on a PANalytical Empyrean powder diffractometer equipped with a PIXcel1D detector (Cu Kα radiation). The operational voltage and current were 40 kV and 40 mA, respectively. The data used for purity identification were collected with the setting of 0.0262/30s. High quality Cu Kα XRD data for Rietveld refinements were collected with the setting of 0.0131/200s for $CaBa_{1-x}Pb_xZn_2Ga_2O_7$ (x = 0-1). The high quality XRD data with Cu $K\alpha_1$ radiation for $CaBa_{1-x}Pb_xZn_2Ga_2O_7$ (x = 0, 0.5, and 1) were collected by using PANalytical Empyrean powder diffractometer equipped with a Ge (111) primary beam monochromator.

Supplementary Note 2. Reciprocal space Rietveld refinement (X-ray and neutron).

The Rietveld refinements against Cu Kα XRD data were performed on all solid solutions $CaBa_{1-x}Pb_xZn_2Ga_2O_7$ and $CaBa_{0.5}Sr_{0.5}Zn_2Ga_2O_7$ by using $CaBaZn_2Ga_2O_7$ as the starting model. There are seven crystallographic sites in this structure model with one for Ca^{2+}, one for Ba^{2+}, two independent sites (T1 and T2) for Zn^{2+}/Ga^{3+}, and three independent sites for oxygen atoms (O1, O2, and O3). As Zn^{2+} and Ga^{3+} could not be distinguished by X-ray, Zn^{2+} and Ga^{3+} were regarded as the same cation during the initial refinement process. After the initial refinement, we found that the average <T-O> bond distance for $T1O_4$ tetrahedra is smaller than that of $T2O_4$ tetrahedra, which indicated T1 site is dominated by Ga^{3+} because Ga^{3+} and Zn^{2+} have large difference in cationic radii in 4-coordination (0.47 Å and 0.60Å for Ga^{3+} and Zn^{2+}, respectively). Thus, we treat the T1 and T2

sites were occupied with Ga^{3+} and Zn^{2+}, respectively.

With increasing the Pb^{2+}-content, an increase of *z*-coordination was observed for Ba^{2+}/Pb^{2+}, indicating Ba^{2+}/Pb^{2+} displaces from the center of dodecahedral cavity due to the stereochemically active lone pair effect of Pb^{2+}. At the microscopic level, only Pb^{2+} should deviate from the center of dodecahedral cavity, while Ba^{2+} should remain in the center, as in the undoped structure. We thus attempt to refine the Ba^{2+} and Pb^{2+} separately with different crystallographic sites. However, the scattering density does not seem to form two well defined minima and unconstrained refinement resulted in Ba^{2+} also drifting from the center of the $[O_{12}]$ dodecahedron. Similar result was observed for the combined refinement for $CaBa_{0.5}Pb_{0.5}Zn_2Ga_2O_7$ against both Cu $K\alpha_1$ XRD and constant wavelength ND data. Therefore, the subsequent refinements for $CaBa_{1-x}Pb_xZn_2Ga_2O_7$ (x = 0.2-0.9) were carried out with Ba^{2+}/Pb^{2+} located at the same site and using anisotropic atomic displacements (ADPs) to account for Ba^{2+}/Pb^{2+} local disordering. A highly anisotropic thermal motion elongated along *c*-axis was observed for Ba^{2+}/Pb^{2+} (Supplementary Table 2), which is in good agreement with the stereochemical effect of Pb^{2+}. For $CaPbZn_2Ga_2O_7$, a flattened ADP was observed for Pb^{2+}, indicating Pb^{2+} deviates from the 3-fold symmetry. Considering that Pb^{2+} deviates from the center of $[PbO_{12}]$ dodecahedron to form strong covalency bond with O1, which is revealed by our theoretical calculations, a splitting model with Pb^{2+} towards to one of the O1 atoms was thus used to account for Pb^{2+} disordering in $CaPbZn_2Ga_2O_7$. The final crystallographic parameters obtained from XRD are summarized in Supplementary Tables 2 and 3.

The structure models obtained from XRD were used as starting model for Combined Rietveld

refinements against both Cu Kα1 data and constant wavelength neutron data of $CaBa_{1-x}Pb_xZn_2Ga_2O_7$ (x = 0, 0.5, and 1). Owing to the large contrast in neutron scattering length between Zn^{2+} (5.68 fm) and Ga^{3+} (7.29 fm), the occupancies of Zn^{2+} and Ga^{3+} at different T-sites could be determined precisely. The precise crystal structure, including atomic occupancies, atomic sited for both heavy (Ba^{2+}/Pb^{2+}) and light atoms (O), thus can be determined by such combined refinements. The final crystallographic parameters obtained from combined Rietveld refinements for $CaBa_{1-x}Pb_xZn_2Ga_2O_7$ (x = 0, 0.5, and 1) are summarized in Supplementary Tables 4 and 5.

Supplementary Note 3. Real space Rietveld refinement.

The local structure of $CaBa_{1-x}Pb_xZn_2Ga_2O_7$ (x = 0, 0.5, 1.0) was investigated via analysis of the real space neutron PDF (pairs distribution function, G(r)) using the TOPAS-Academic V6 software. Initial small box PDF refinements using the $P6_3mc$ structure model extracted from reciprocal space Rietveld refinements led to large discrepance between the observed and calculated patterns, especially for Ca–O and Zn/Ga–O atomic pairs. Then refinements were performed with the $P31c$ and $Pna2_1$ models, which was derived with the $P6_3mc$ aristotype structure using the ISODISTORT software suit.[1] Due to the relative complexity of the $Pna2_1$ model, in comparison with $P31c$ and $P6_3mc$ structure models, simulated annealing method was employed to determine the atomic coordinates precisely during the PDF refinements process. The refinements using $P6_3mc$ and $P31c$ models yield similar reliable factors. In contrast, both the reliable factors and the fits of Ca–O and Zn/Ga–O atomic pairs were improved significantly by using the $Pna2_1$ model, indicating

the local structure symmetry of $CaBa_{1-x}Pb_xZn_2Ga_2O_7$ should be *Pna*2_1.

In the *P*6_3*mc* and *P*31*c* models, O1, O3, and T2 atoms are located at the 6*c* sites. In the microscopic structure, the 3-fold axis should be not retained duo to the Zn^{2+}/Ga^{3+} disordering in T2 sites. Additionally, owing to the large difference in cationic radius between Zn^{2+} and Ga^{3+}, the differences in distance between Zn–O and Ga–O pairs also drives O1 atoms (and O3 atoms) deviate from 3-fold axis symmetry. In other words, O1 atoms (O3 atoms, and T2 site atoms) are not strictly related by the 3-fold axis in the local structure because of the Zn^{2+}/Ga^{3+} disordering and large difference in cationic size. In contrast, in the *Pna*2_1 model, there are 7 symmetry-independent oxygens and 4 symmetry-independent T-site atoms (Zn/Ga) to describe the local structural distortion, due to absence of 3-fold axis symmetry. Finally, we can conclude that the local structure distortion observed in $CaBa_{1-x}Pb_xZn_2Ga_2O_7$ is not induced by Pb-doping but Zn^{2+}/Ga^{3+} disordering. As indicated by our real space refinements, such Zn^{2+}/Ga^{3+} disordering induced local structural distortions could be readily detected by nPDFs.

Supplementary References.

1. Campbell, B. J.; Stokes, H. T.; Tanner, D. E.; Hatch, D. M. *J. Appl. Cryst.* **39**, 607−614 (2006).
2. Errandonea, D., Pellicer-Porres, J., Manjon, F. J., Segura, A., Ferrer-Roca, C., Kumar, R. S., Tschauner, O., Lopez-Solano, J., Rodriguez-Hernandez, P., Radescu, S., Mujica, A., Munoz, A., Aquilanti, G. *Phys. Rev. B: Condens. Matter.* **73**, 224103 (2006).
3. Stoltzfus, M. W., Woodward, P. M., Seshadri, R., Klepeis, J., Bursten, B. *Inorg. Chem.* **46**, 3839−3850, (2007).
4. Mueller Buschbaum, H., Deiseroth, H. J. *J. Inorg. Nucl. Chem.* **35**, 3177−3182, (1973).
5. Bernal, I., Marsh, R. E. *Z. Anorg. Allg. Chem.* **488**, 38−44, (1982).
6. Chaudhurya, S., Rakshit, S. K., Parida, S. C., Singh, Z., Singh-Mudher, K. D., Venugopal, V. *J Alloy. Compd.* **455**, 25−30, (2008).
7. Ma, H. Q., Lin K., Fan, L. L., Rong, Y.C, Chen, J., Deng, J. X, Liu, L. J., Kawaguchi, S., Katoe, K. Xing, X. R. *RSC Adv.* **5**, 71890, (2015).
8. Lin, K., Zhou, Z. Y., Liu, L. J., Ma, H. Q., Chen, J., Deng, J. X., Sun, J. L., You, L., Kasai, H., Kato, K., Takata, M., Xing, X. R. *J. Am. Chem. Soc.* **137**, 13468−13471, (2015).
9. Galasso, F., Layden, G., Ganung, G. *Mater. Res. Bull.* **3**, 397−408 (1968).
10. Labbe, P., Frey, M., Raveau, B., Monier, J. C. *Acta Crystallogr.* B33, 2201−2212, (1977).
11. Manoun, B., Popovic, L., de Waal, D., Verryn, S. M. C. *Powder Diffr.* **18**, 122−127 (2003).
12. Angel, R. J., Bismayer, U., Marshall, W.G., *J. Phys: Condens. Matter*. **13**, 5353−5364 (2001).
